# Supplementary figures and images for: Interplay between cohesin and TORC1 links chromosome segregation and gene expression to environmental changes
Source: eLife. 2026 Jun 1;14:RP108275. doi: 10.7554/eLife.108275 (PMC13225845; doi:10.7554/eLife.108275)

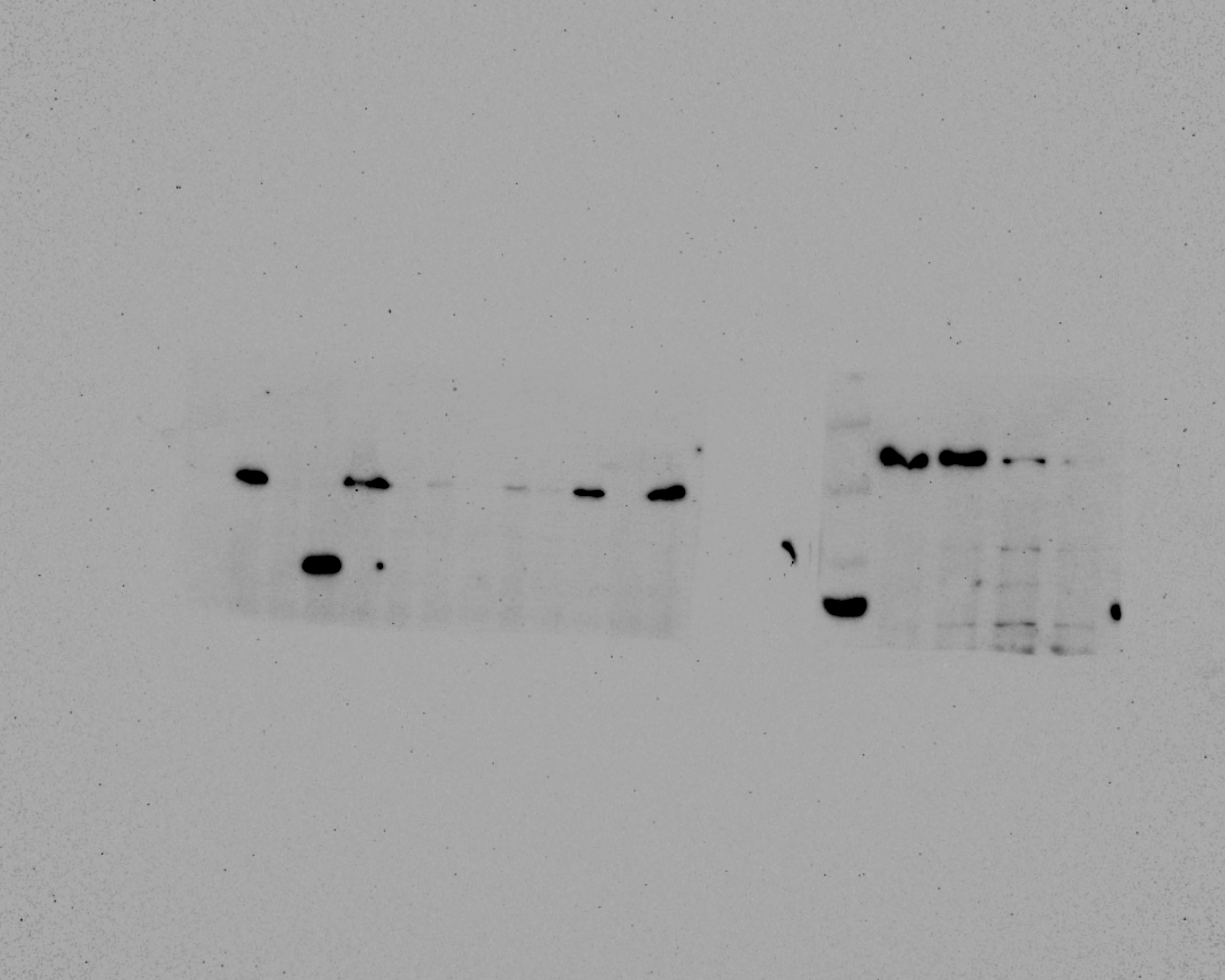

Supplement: Figure 1—source data 1. [file elife-108275-fig1-data1.zip › Figure 1-source data 1/2023-04-13 12h41m14s(Chemiluminescence).tif]

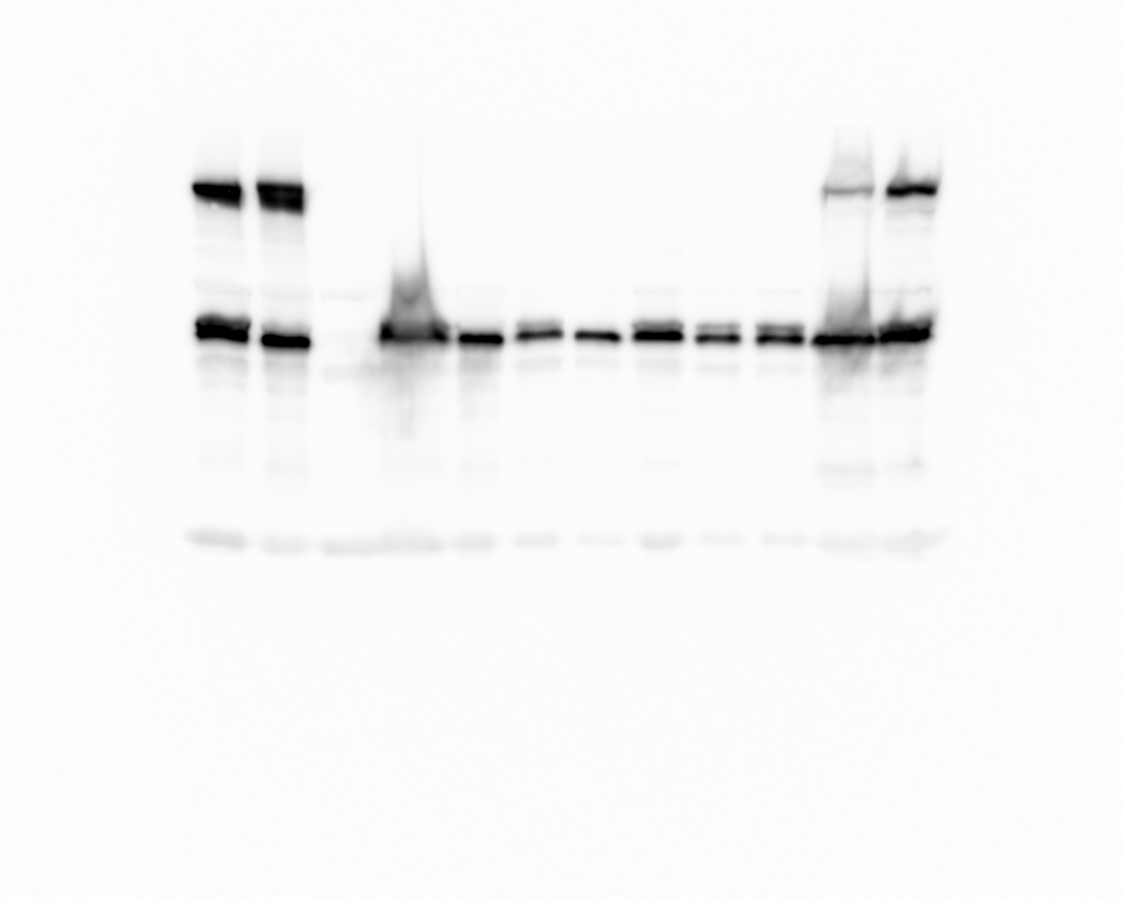

Supplement: Figure 1—source data 1. [file elife-108275-fig1-data1.zip › Figure 1-source data 1/2023-04-14 11h27m07s(Chemiluminescence).tif]

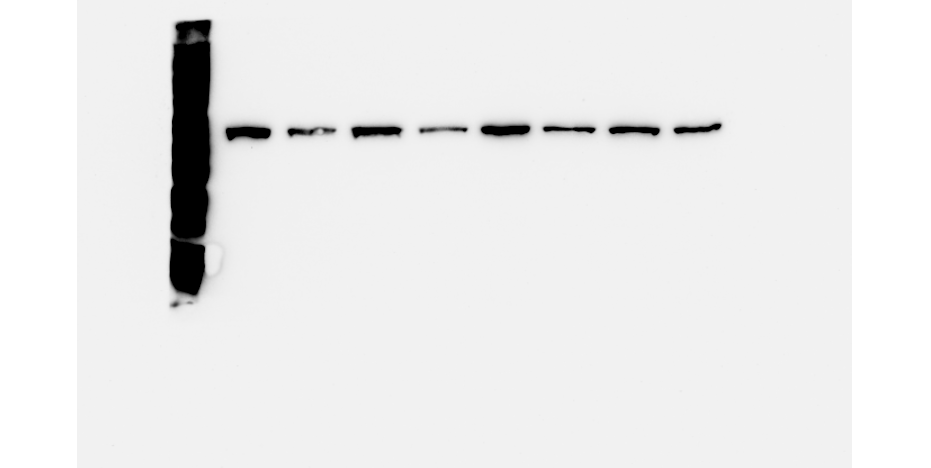

Supplement: Figure 2—figure supplement 3—source data 1. [file elife-108275-fig2-figsupp3-data1.zip › Figure 2-figure supplement 3-source data 1/anti-Pms3.tif]

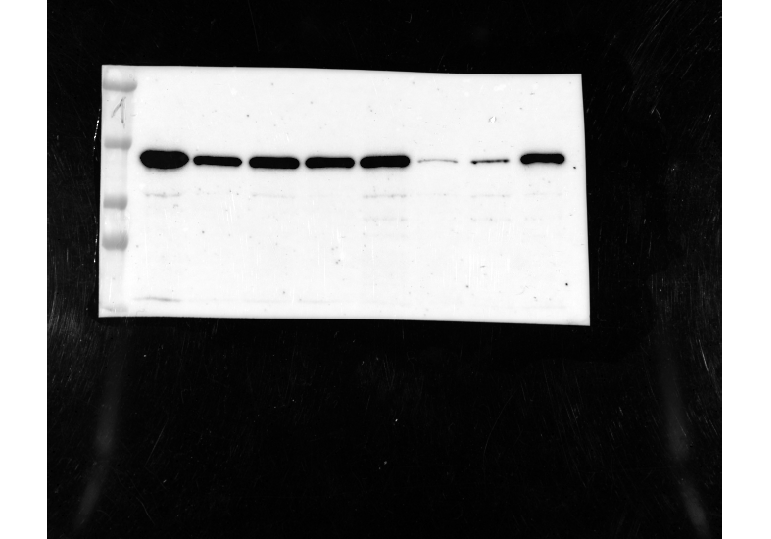

Supplement: Figure 2—figure supplement 3—source data 1. [file elife-108275-fig2-figsupp3-data1.zip › Figure 2-figure supplement 3-source data 1/anti-psm3-K106ac.tif]

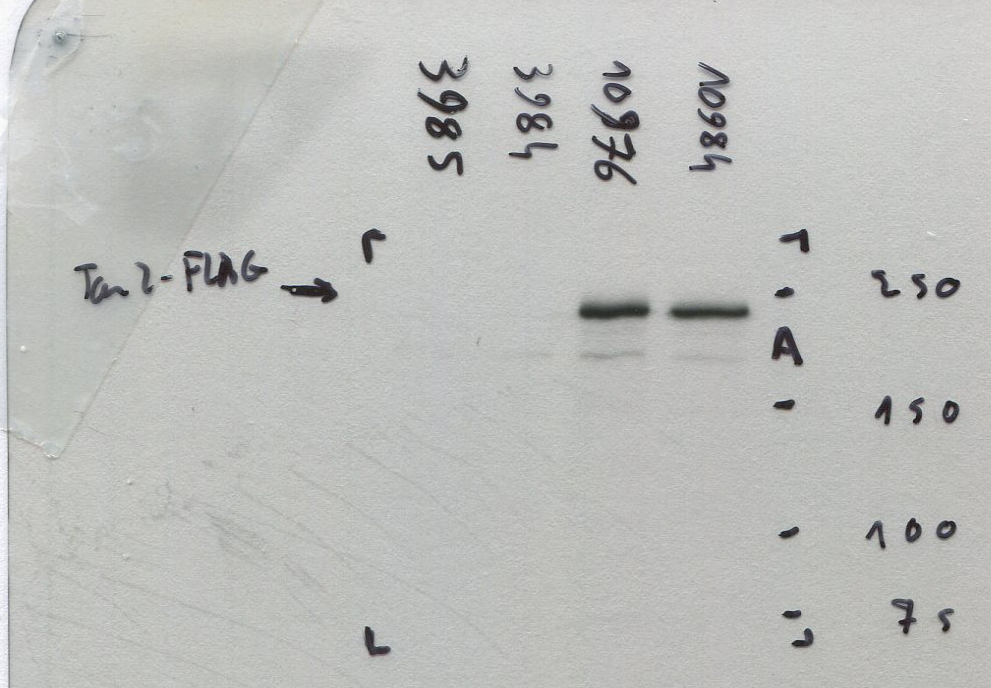

Supplement: Figure 4—source data 1. [file elife-108275-fig4-data1.zip › Figure 4-source data 1/Figure 4B_INPUT_blot FLAG.tif]

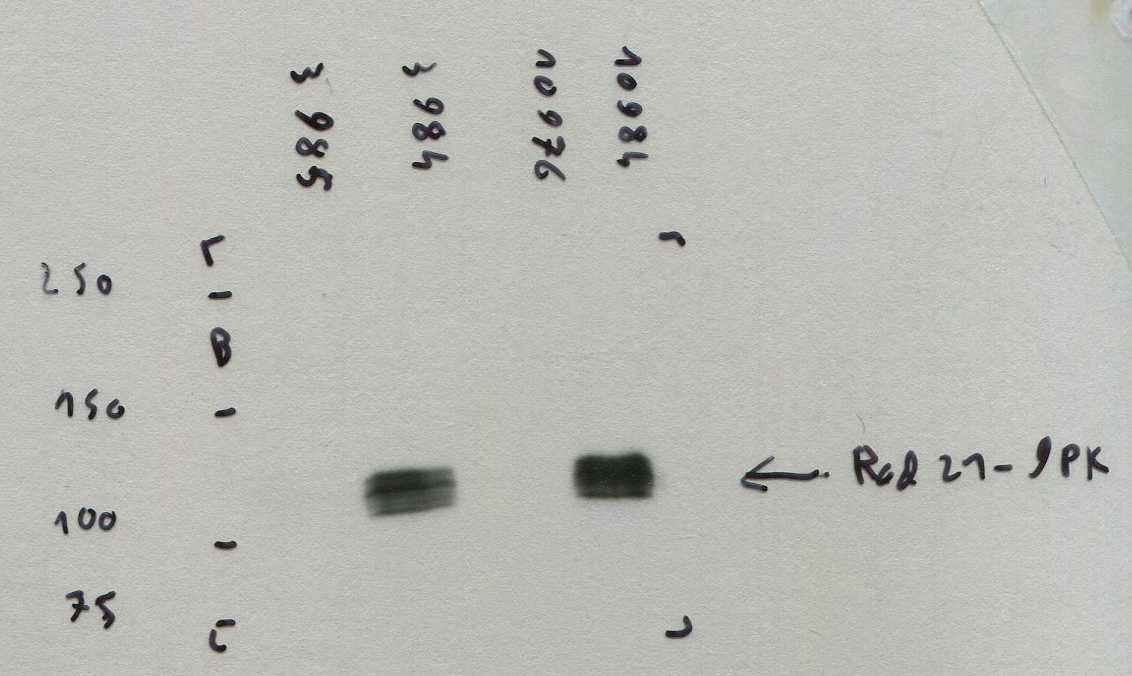

Supplement: Figure 4—source data 1. [file elife-108275-fig4-data1.zip › Figure 4-source data 1/Figure 4B_INPUT_blot PK.tif]

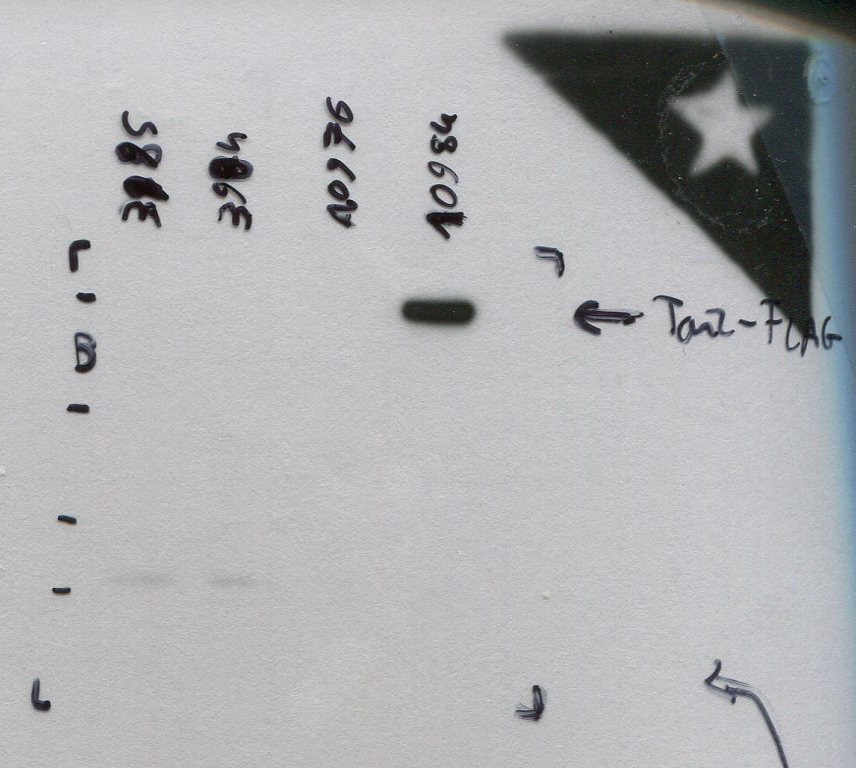

Supplement: Figure 4—source data 1. [file elife-108275-fig4-data1.zip › Figure 4-source data 1/Figure 4B_IP PK_blot FLAG.tif]

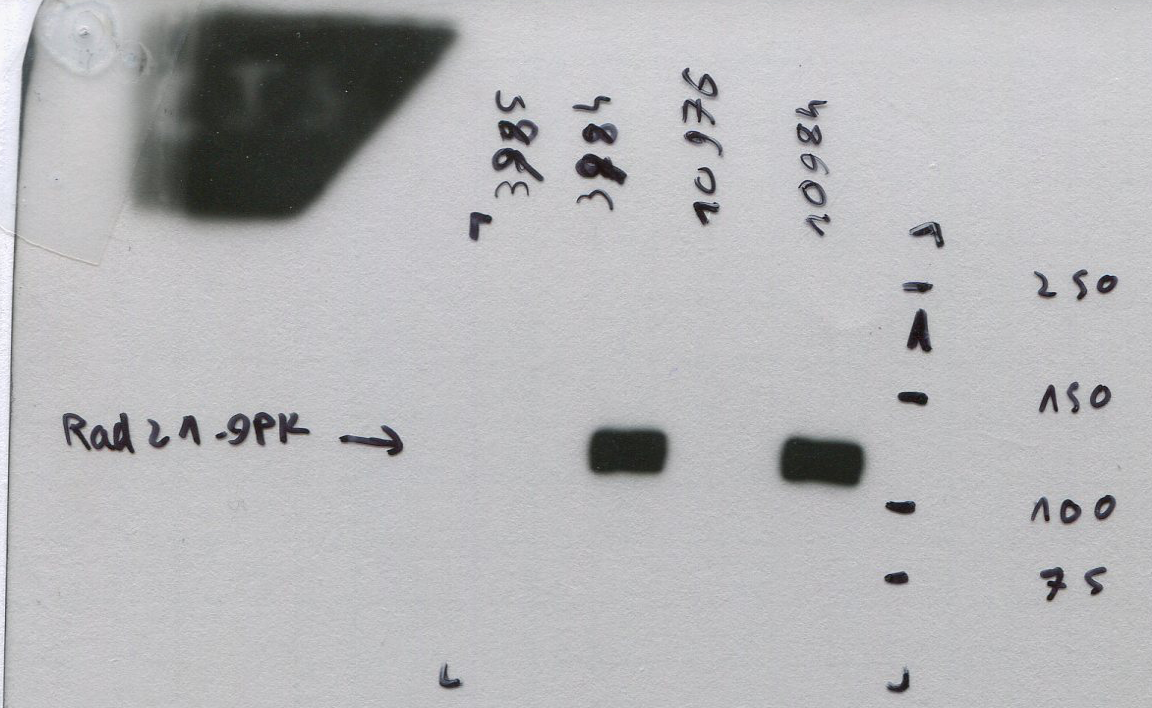

Supplement: Figure 4—source data 1. [file elife-108275-fig4-data1.zip › Figure 4-source data 1/Figure 4B_IP PK_blot PK.tif]

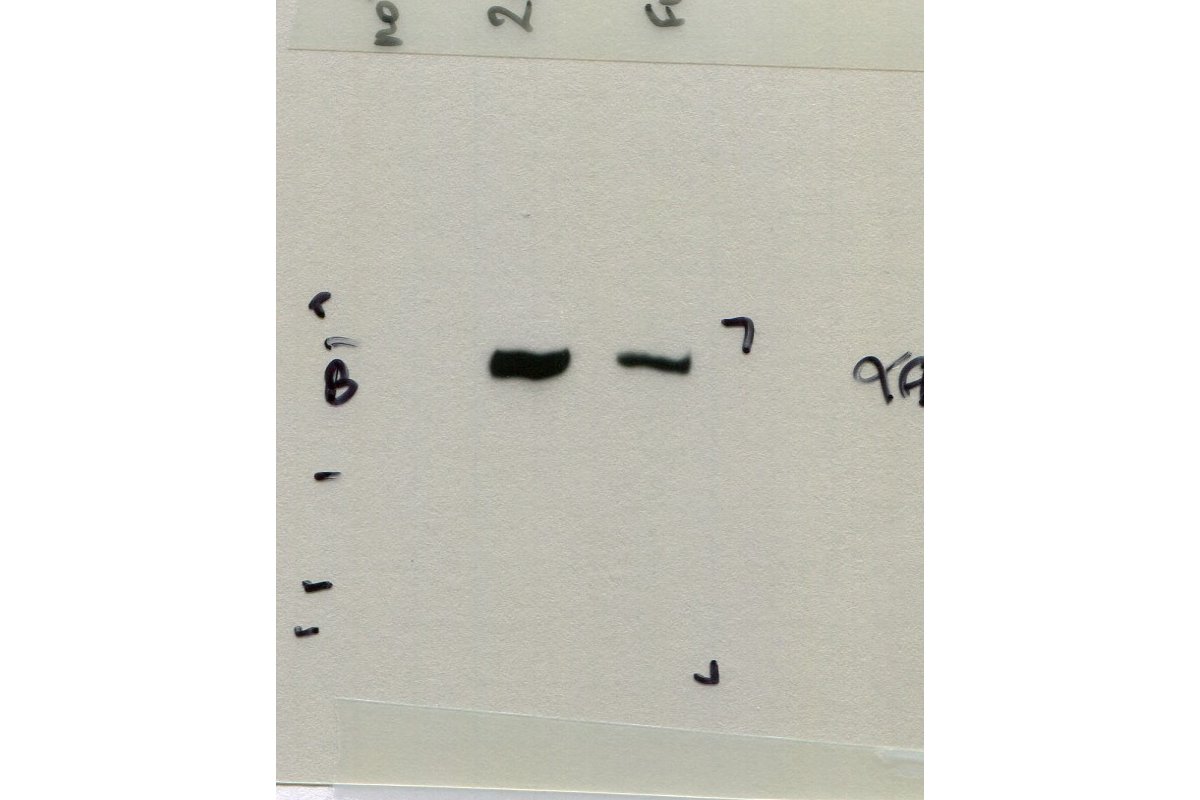

Supplement: Figure 4—source data 1. [file elife-108275-fig4-data1.zip › Figure 4-source data 1/Figure 4C-left-INPUT-blot FLAG.jpg]

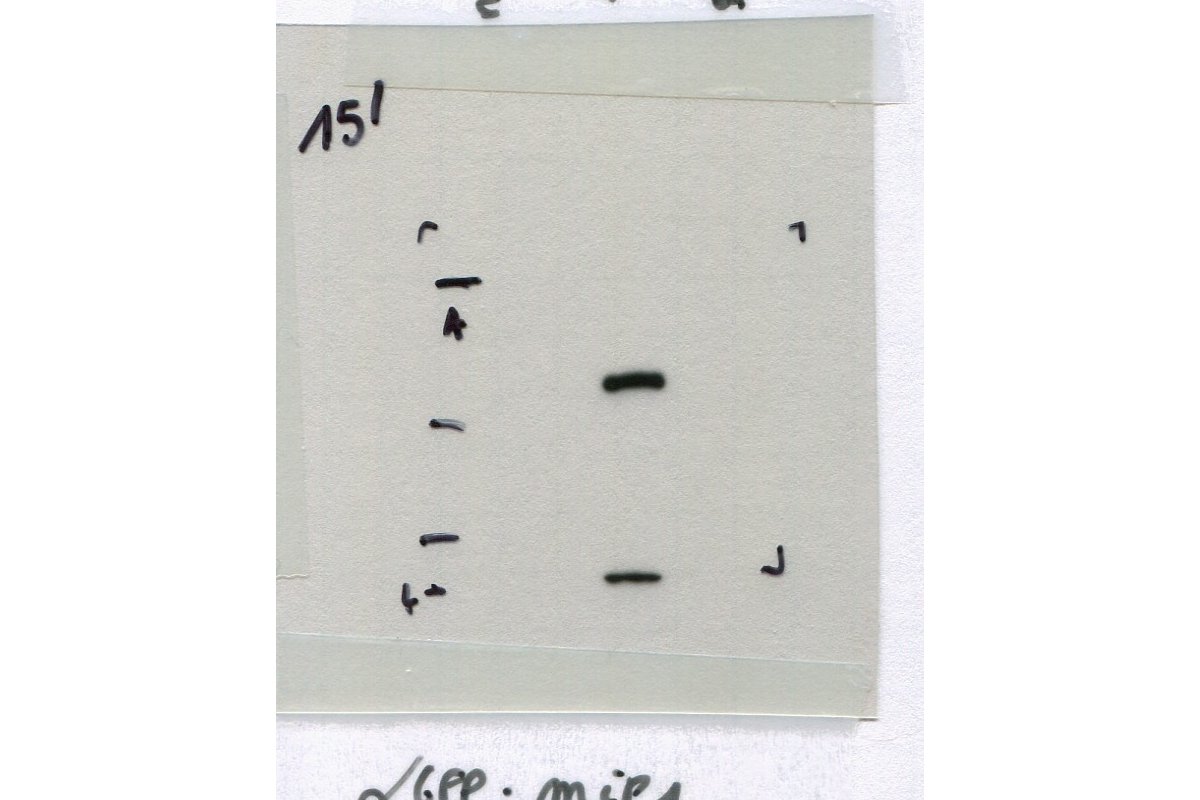

Supplement: Figure 4—source data 1. [file elife-108275-fig4-data1.zip › Figure 4-source data 1/Figure 4C-left-INPUT-blot GFP.jpg]

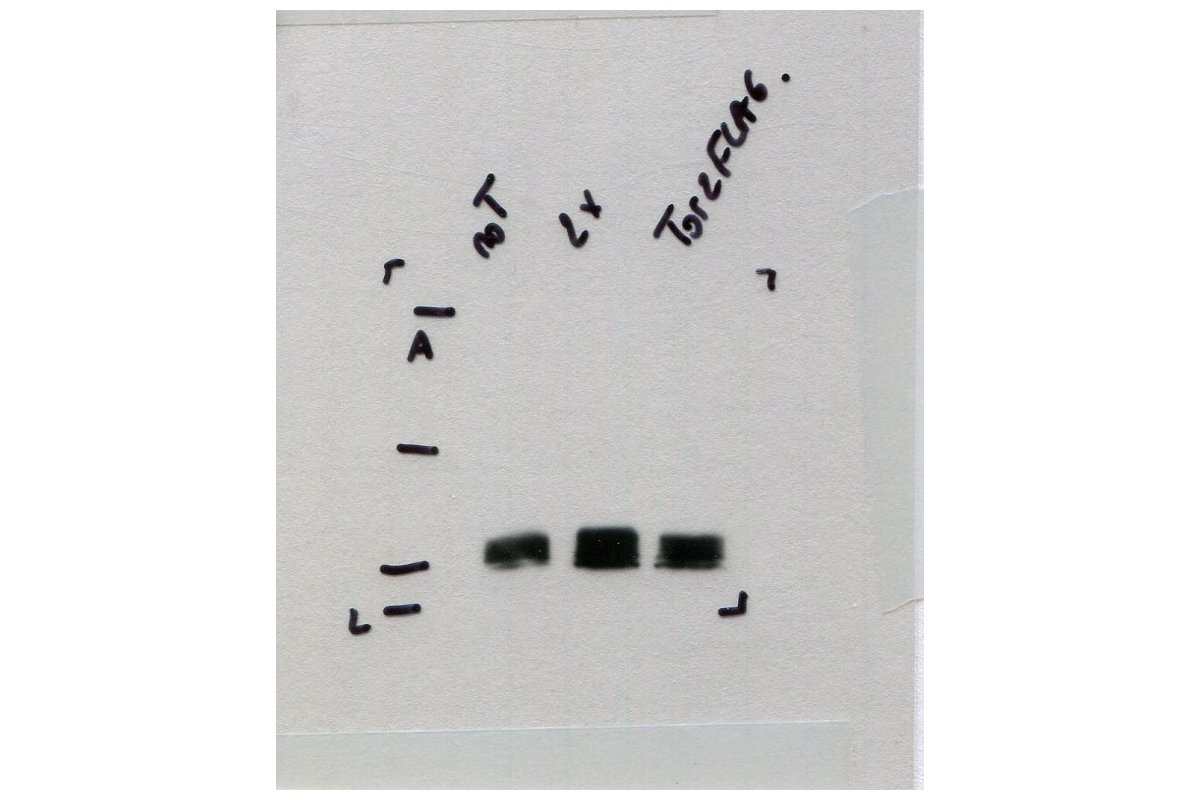

Supplement: Figure 4—source data 1. [file elife-108275-fig4-data1.zip › Figure 4-source data 1/Figure 4C-left-INPUT-blot Rad21.jpg]

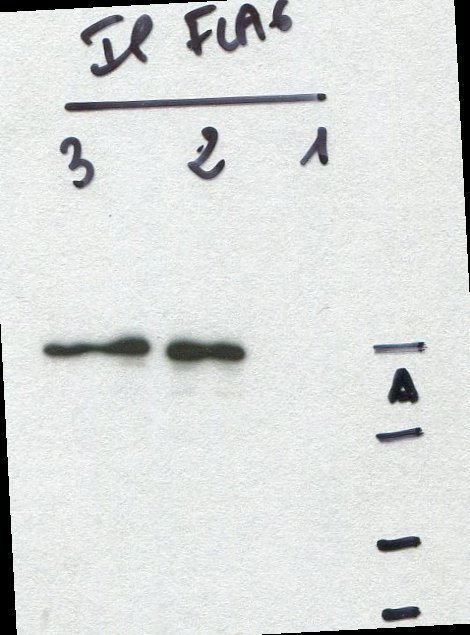

Supplement: Figure 4—source data 1. [file elife-108275-fig4-data1.zip › Figure 4-source data 1/Figure 4C-left-IP FLAG-blot FLAG.jpg]

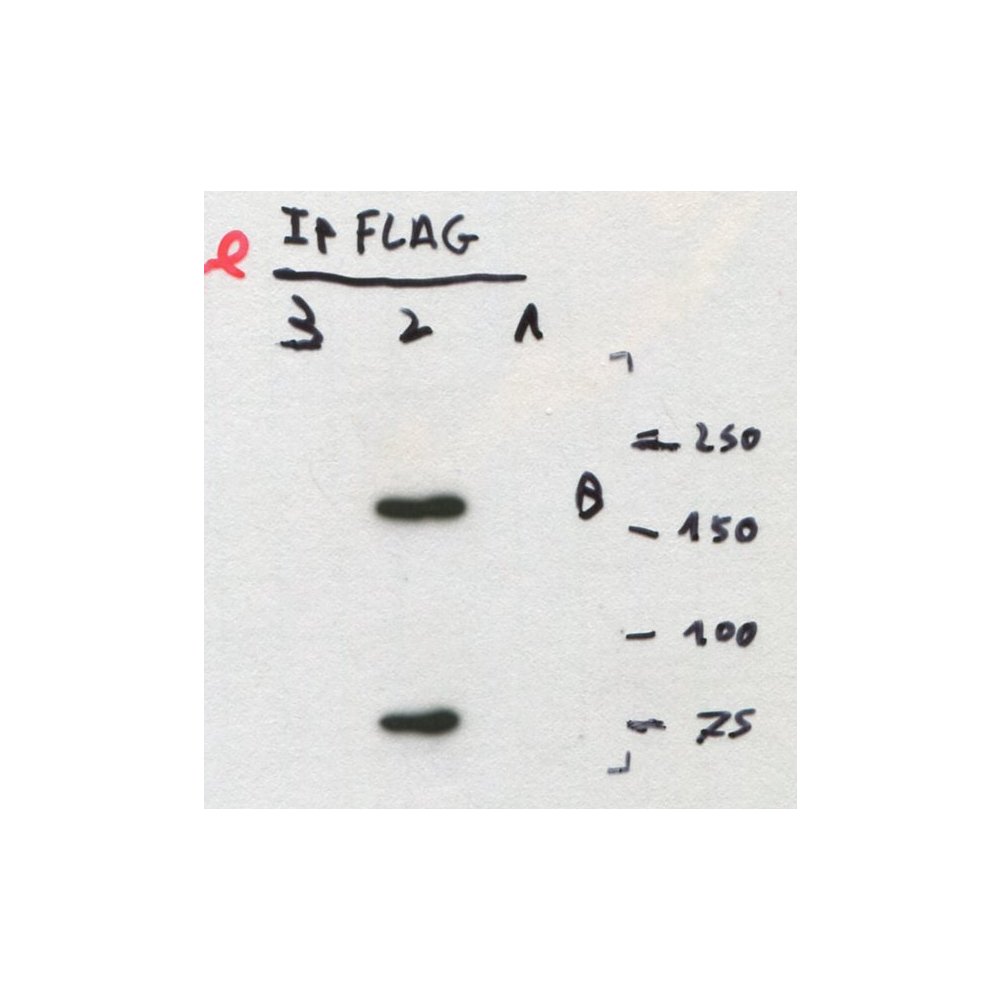

Supplement: Figure 4—source data 1. [file elife-108275-fig4-data1.zip › Figure 4-source data 1/Figure 4C-left-IP FLAG-blot GFP.jpg]

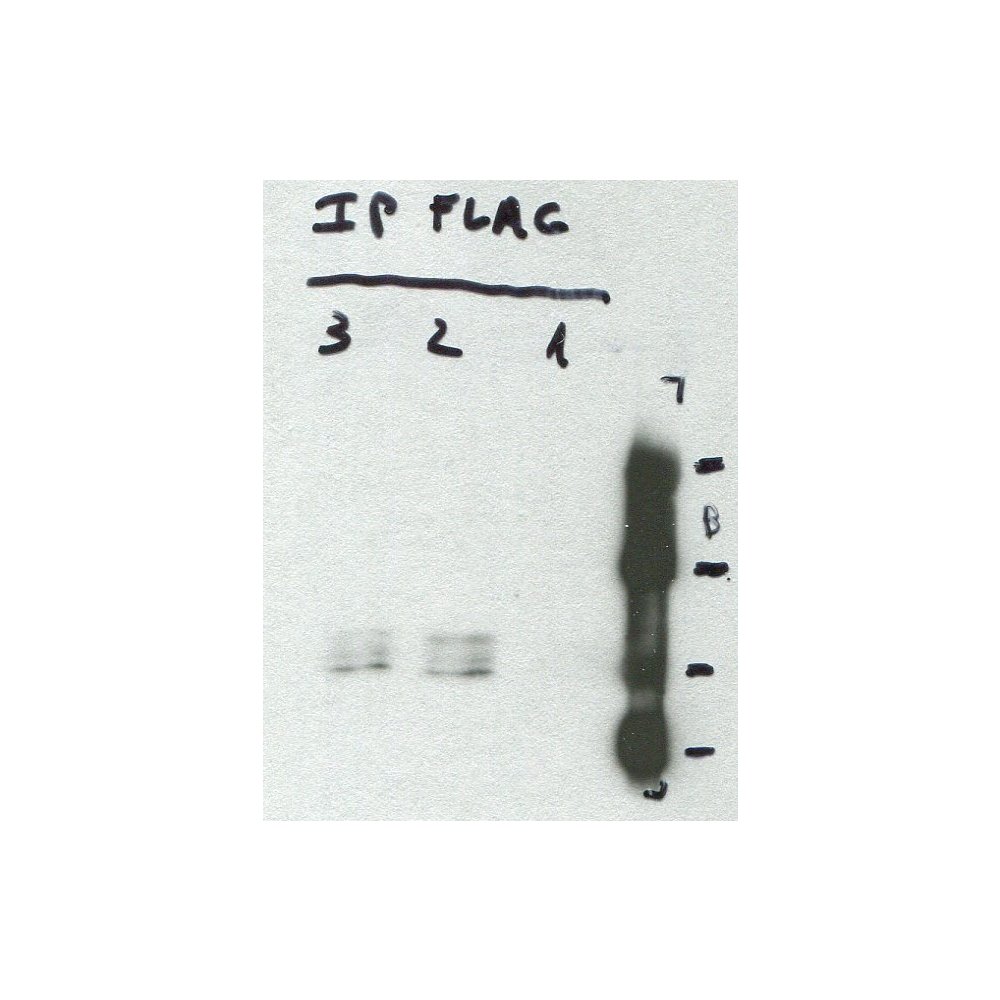

Supplement: Figure 4—source data 1. [file elife-108275-fig4-data1.zip › Figure 4-source data 1/Figure 4C-left-IP FLAG-blot Rad21.jpg]

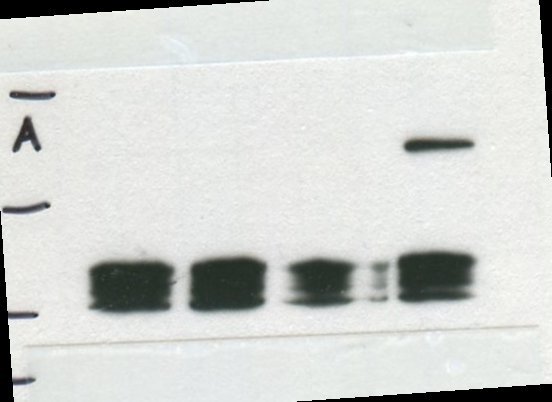

Supplement: Figure 4—source data 1. [file elife-108275-fig4-data1.zip › Figure 4-source data 1/Figure 4C-right-INPUT-blot GFP&Rad21.jpg]

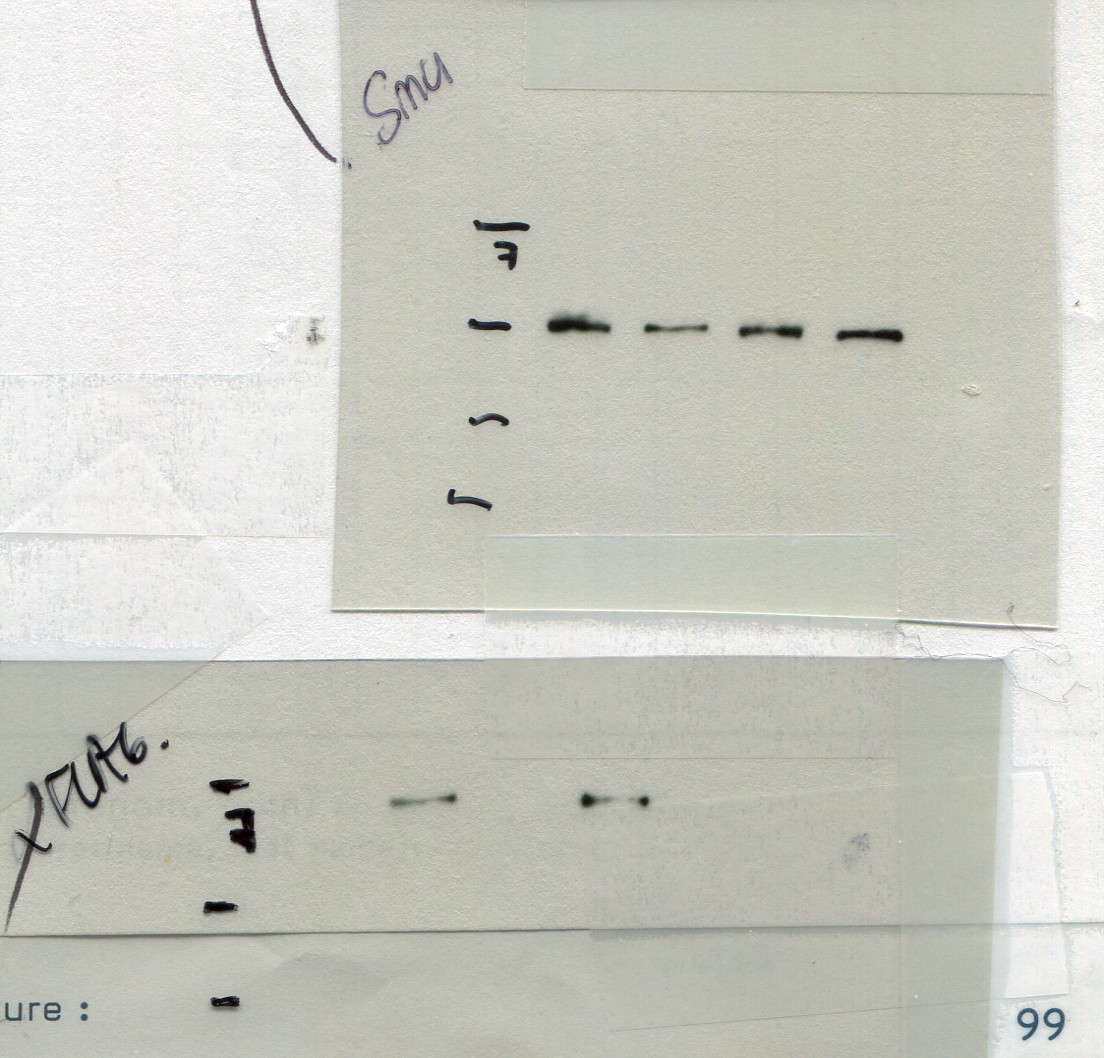

Supplement: Figure 4—source data 1. [file elife-108275-fig4-data1.zip › Figure 4-source data 1/Figure 4C-right-INPUT-blot Psm1&FLAG.jpg]

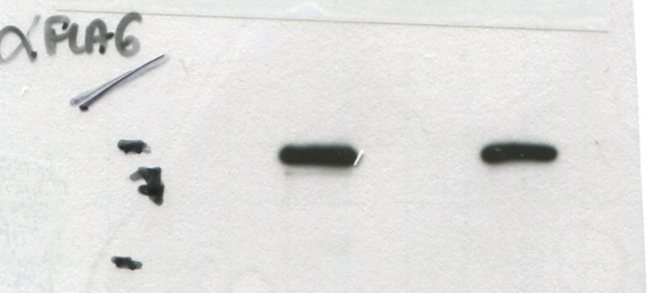

Supplement: Figure 4—source data 1. [file elife-108275-fig4-data1.zip › Figure 4-source data 1/Figure 4C-right-IP FLAG-blot FLAG.jpg]

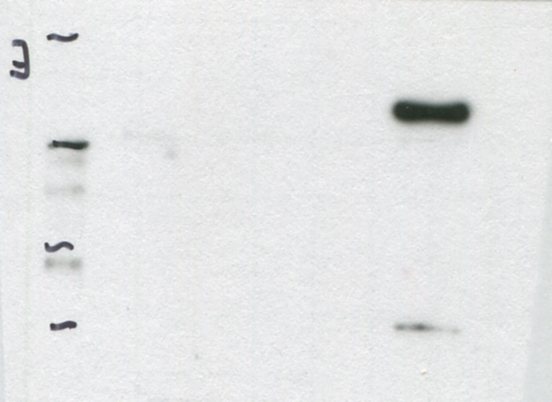

Supplement: Figure 4—source data 1. [file elife-108275-fig4-data1.zip › Figure 4-source data 1/Figure 4C-right-IP-blot GFP.jpg]

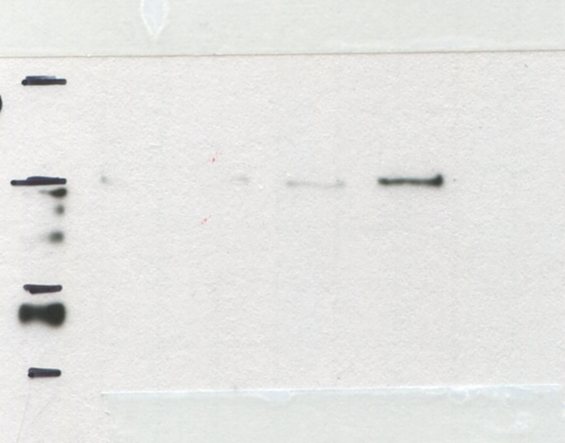

Supplement: Figure 4—source data 1. [file elife-108275-fig4-data1.zip › Figure 4-source data 1/Figure 4C-right-IP-blot Psm1.jpg]

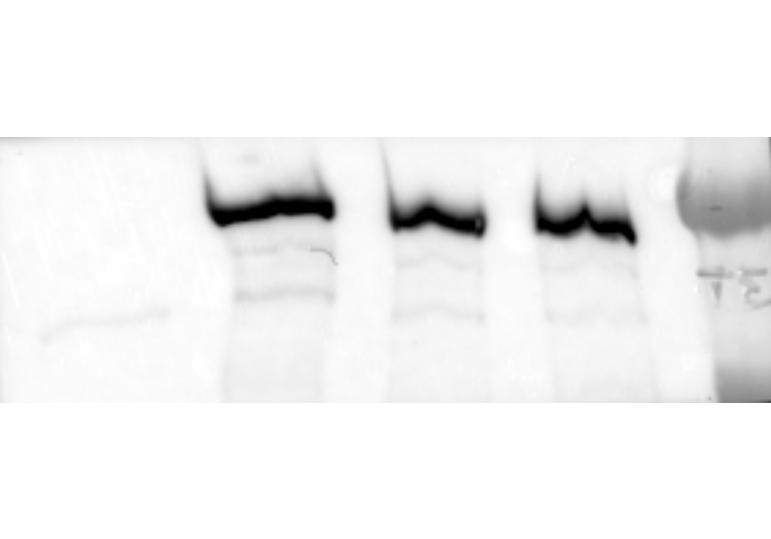

Supplement: Figure 4—figure supplement 1—source data 1. [file elife-108275-fig4-figsupp1-data1.zip › Figure 4-figure supplement 1-source data 1/Panel B_INPUT-Blot FLAG.tif]

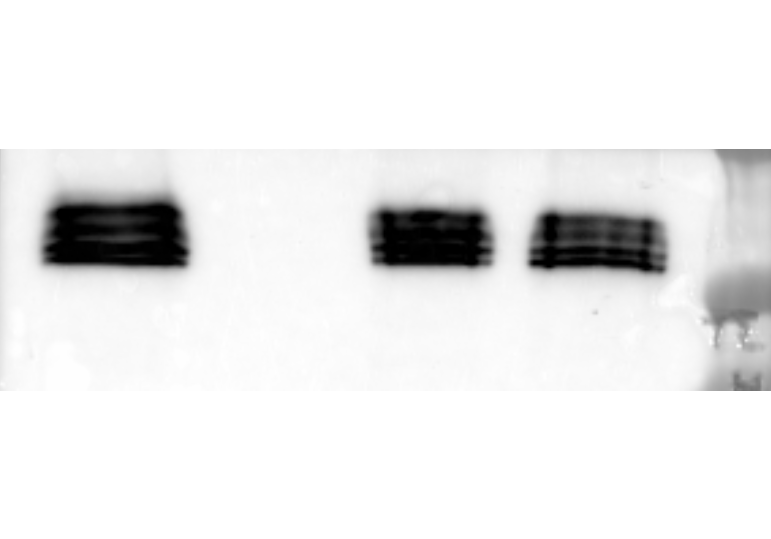

Supplement: Figure 4—figure supplement 1—source data 1. [file elife-108275-fig4-figsupp1-data1.zip › Figure 4-figure supplement 1-source data 1/Panel B_INPUT-Blot PK.tif]

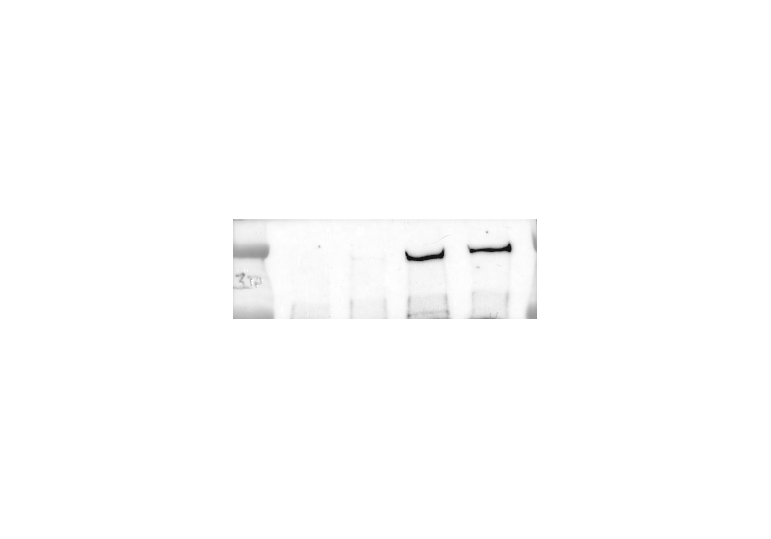

Supplement: Figure 4—figure supplement 1—source data 1. [file elife-108275-fig4-figsupp1-data1.zip › Figure 4-figure supplement 1-source data 1/Panel B_IP PK-Blot FLAG.tif]

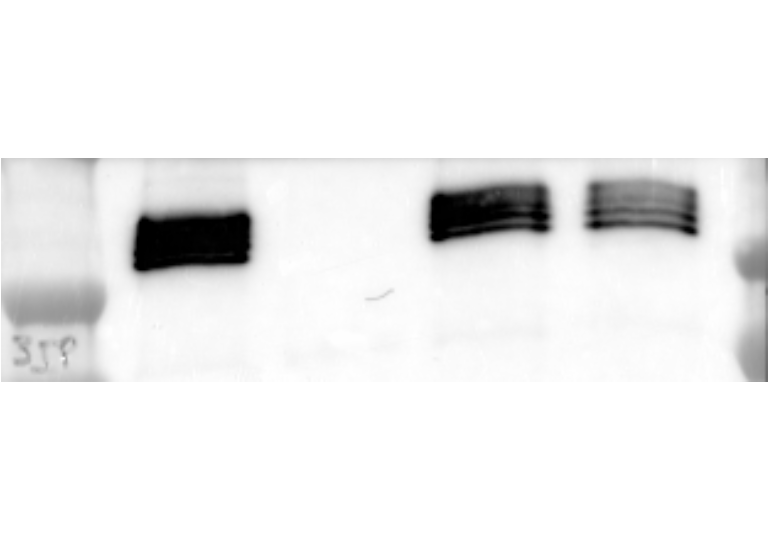

Supplement: Figure 4—figure supplement 1—source data 1. [file elife-108275-fig4-figsupp1-data1.zip › Figure 4-figure supplement 1-source data 1/Panel B_IP PK-Blot PK.tif]

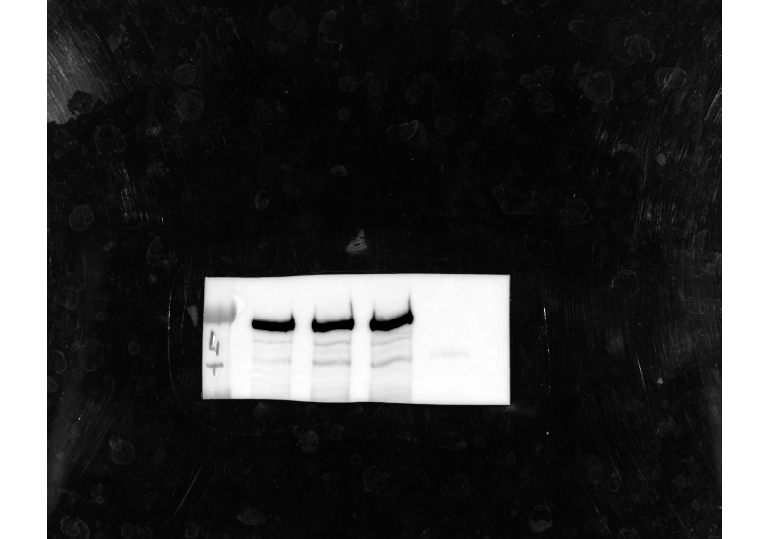

Supplement: Figure 4—figure supplement 1—source data 1. [file elife-108275-fig4-figsupp1-data1.zip › Figure 4-figure supplement 1-source data 1/Panel C_INPUT-Blot FLAG.tif]

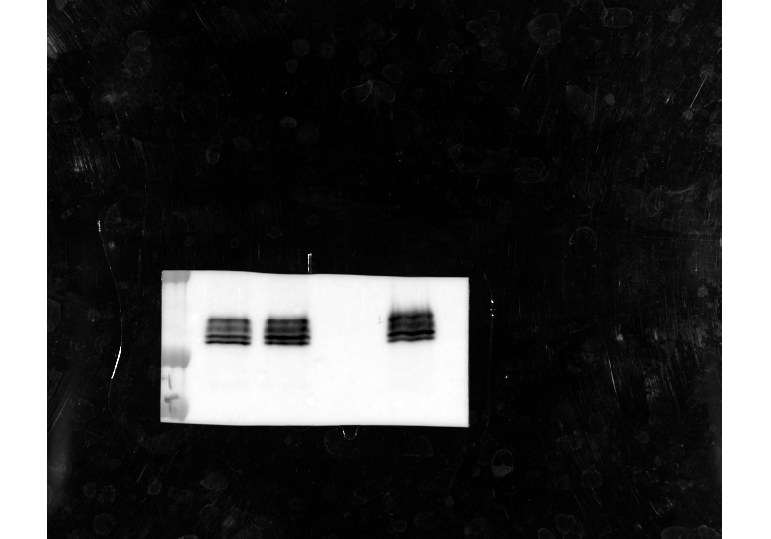

Supplement: Figure 4—figure supplement 1—source data 1. [file elife-108275-fig4-figsupp1-data1.zip › Figure 4-figure supplement 1-source data 1/Panel C_INPUT-Blot PK.tif]

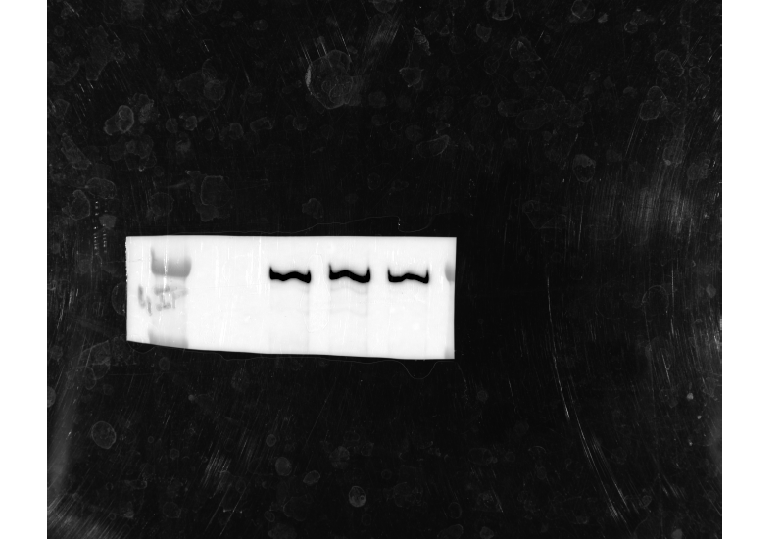

Supplement: Figure 4—figure supplement 1—source data 1. [file elife-108275-fig4-figsupp1-data1.zip › Figure 4-figure supplement 1-source data 1/Panel C_IP FLAG-Blot FLAG.tif]

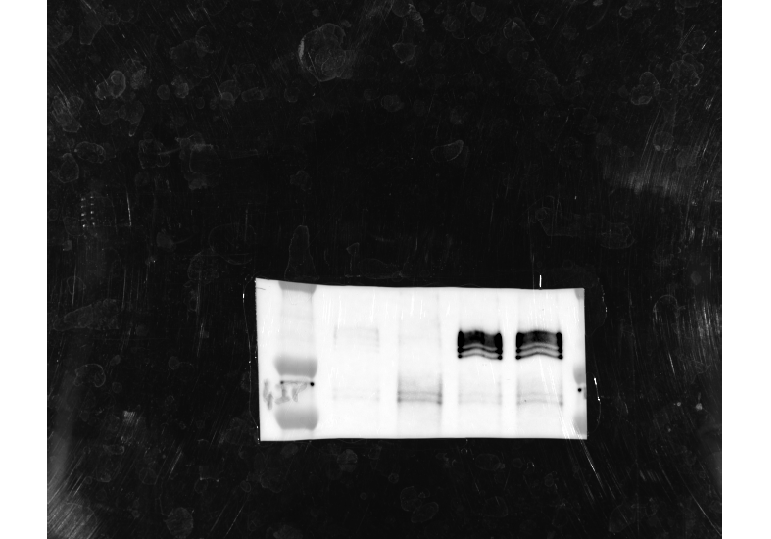

Supplement: Figure 4—figure supplement 1—source data 1. [file elife-108275-fig4-figsupp1-data1.zip › Figure 4-figure supplement 1-source data 1/Panel C_IP FLAG-Blot PK.tif]

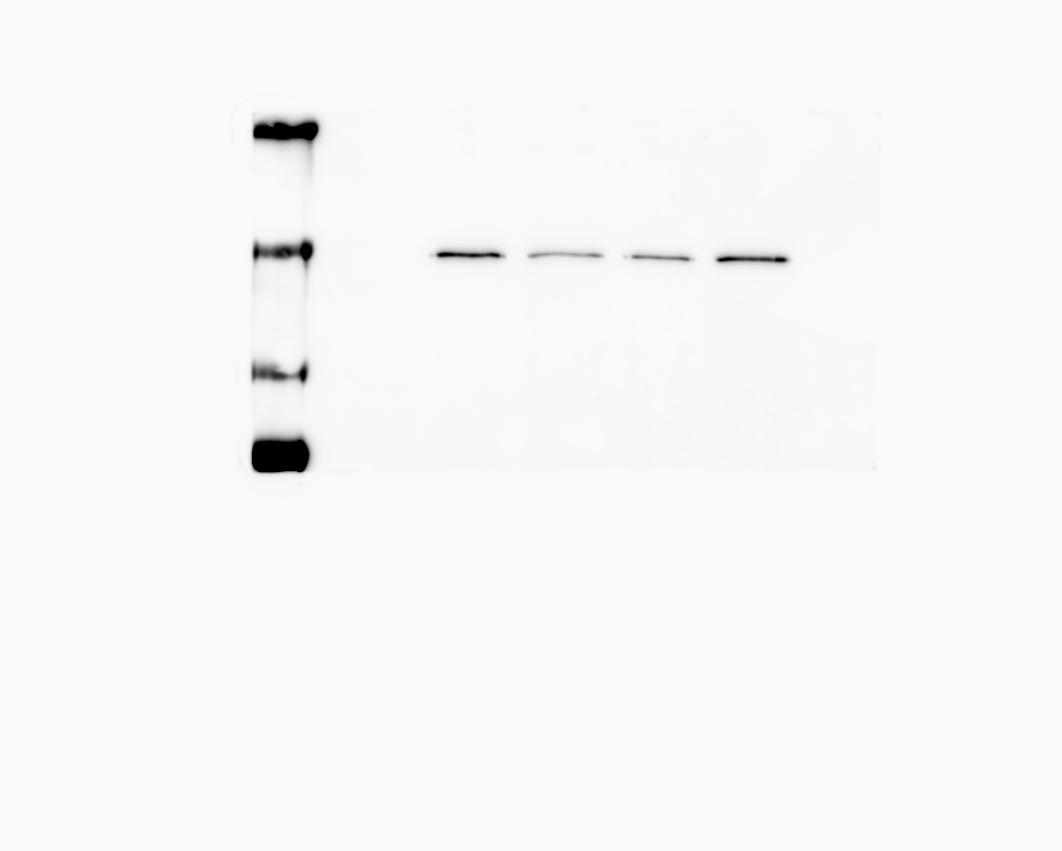

Supplement: Figure 5—source data 1. [file elife-108275-fig5-data1.zip › Figure 5-source data 1/Figure 5B_anti-Psm1-S1022p.tif]

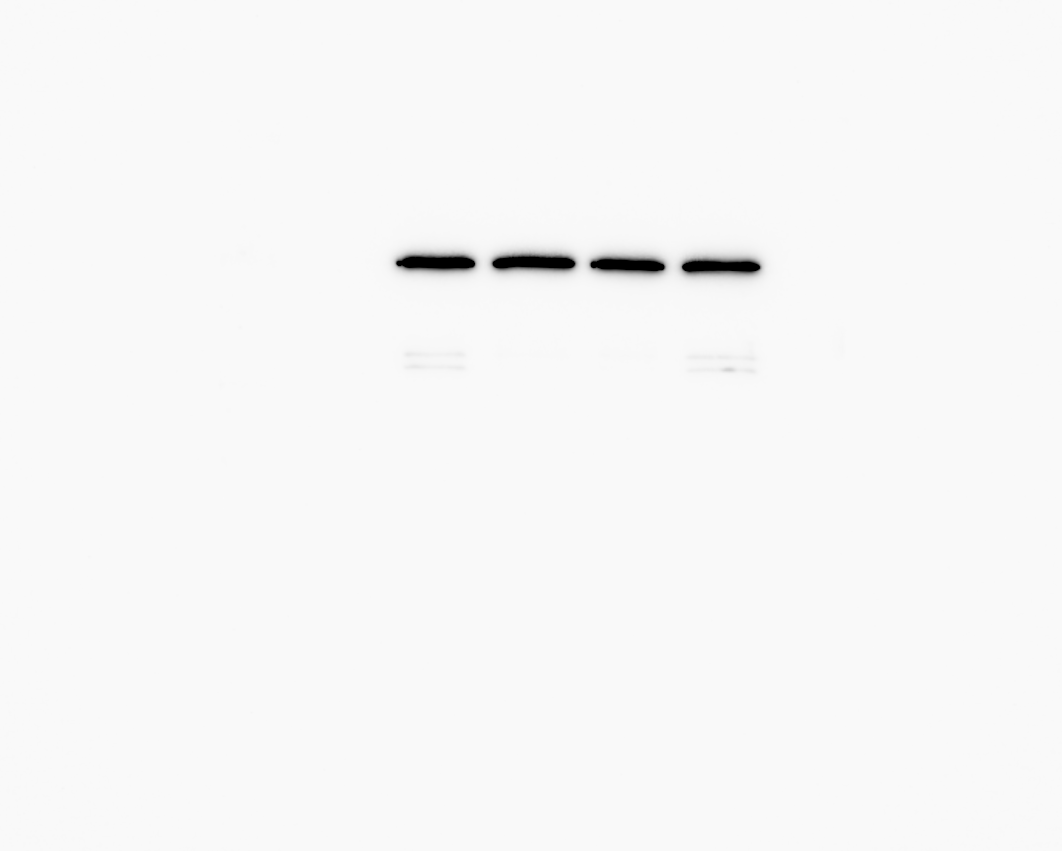

Supplement: Figure 5—source data 1. [file elife-108275-fig5-data1.zip › Figure 5-source data 1/Figure 5B_anti-Psm1.tif]

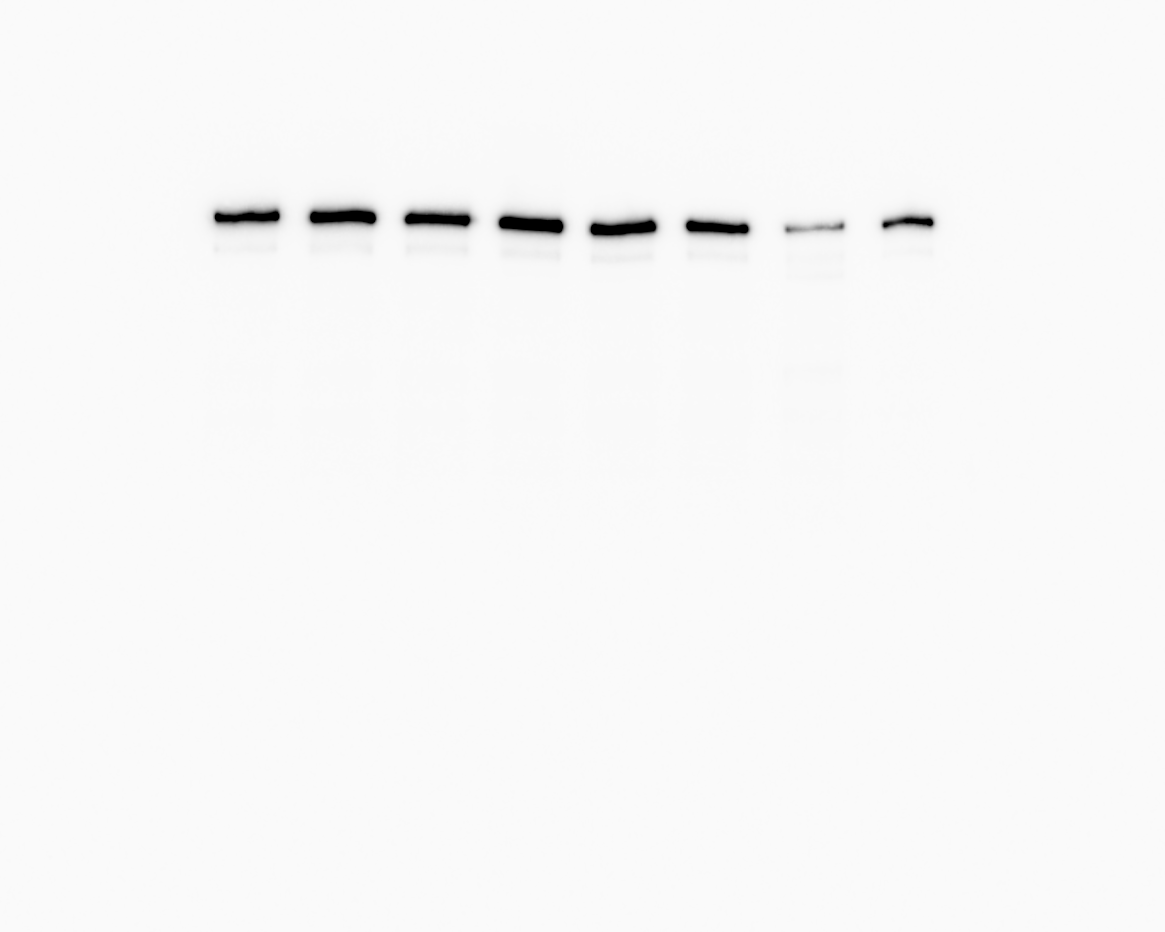

Supplement: Figure 5—source data 1. [file elife-108275-fig5-data1.zip › Figure 5-source data 1/Figure 5C_anti-GFP.tif]

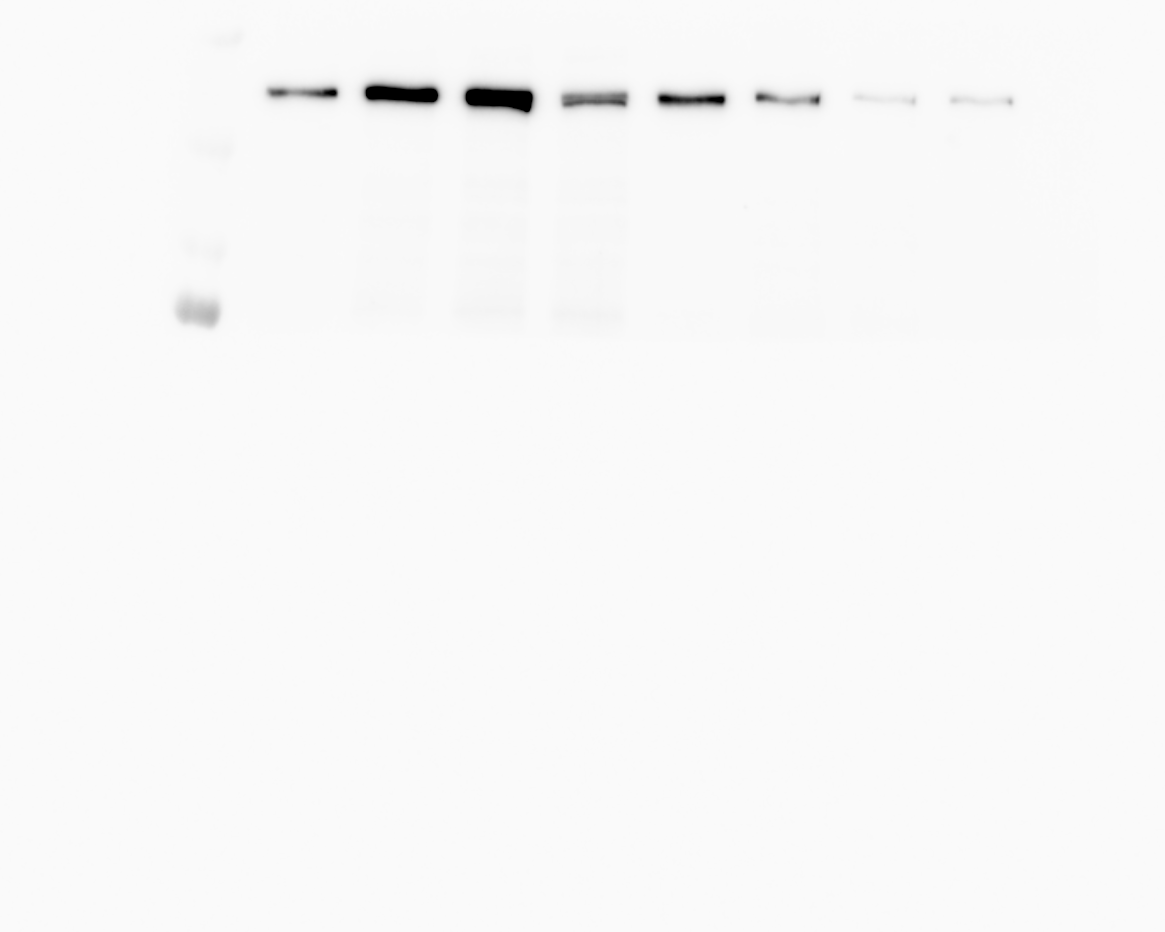

Supplement: Figure 5—source data 1. [file elife-108275-fig5-data1.zip › Figure 5-source data 1/Figure 5C_anti-Mis4-S183p.tif]

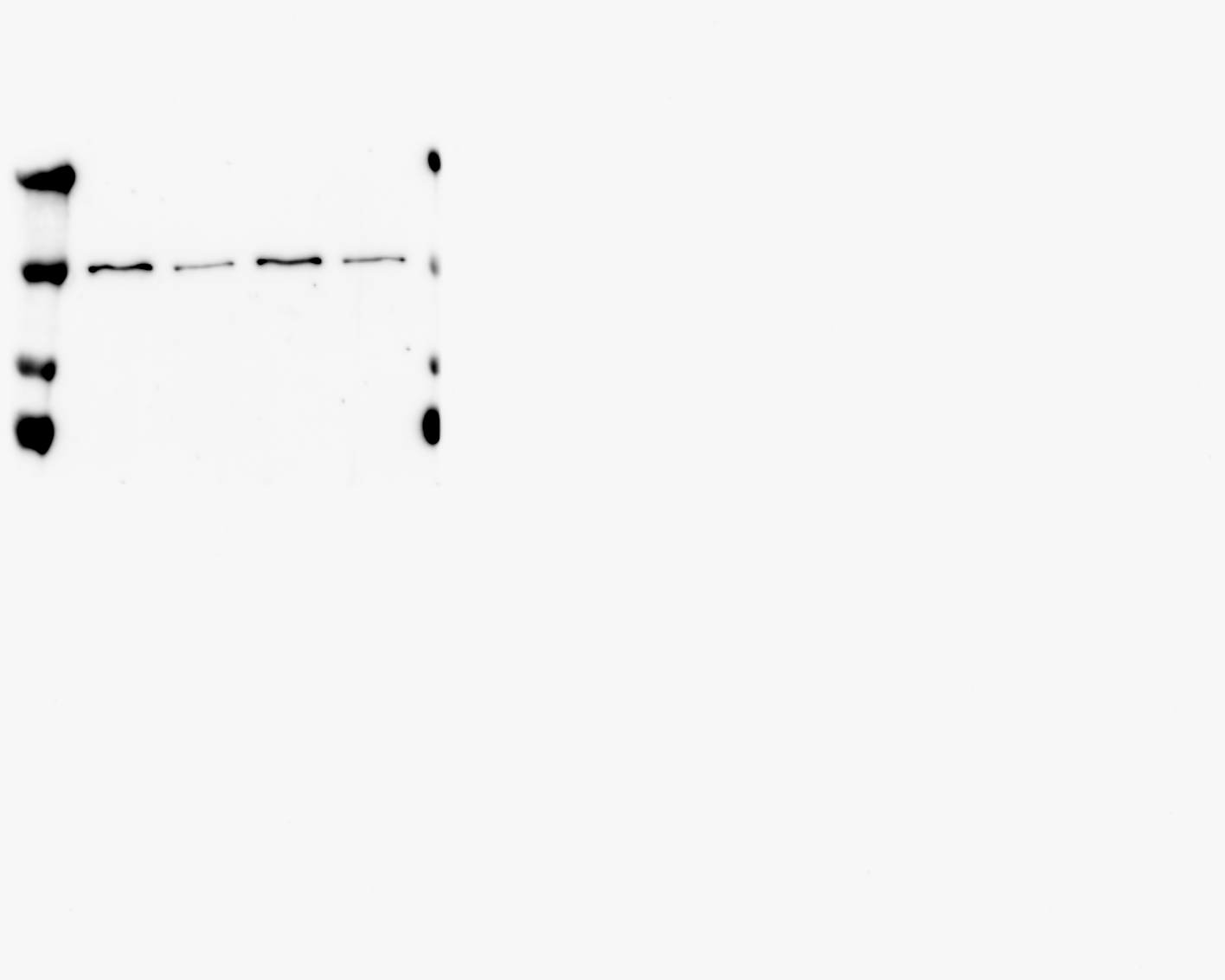

Supplement: Figure 5—source data 1. [file elife-108275-fig5-data1.zip › Figure 5-source data 1/Figure 5D_anti-Psm1-S1022p.tif]

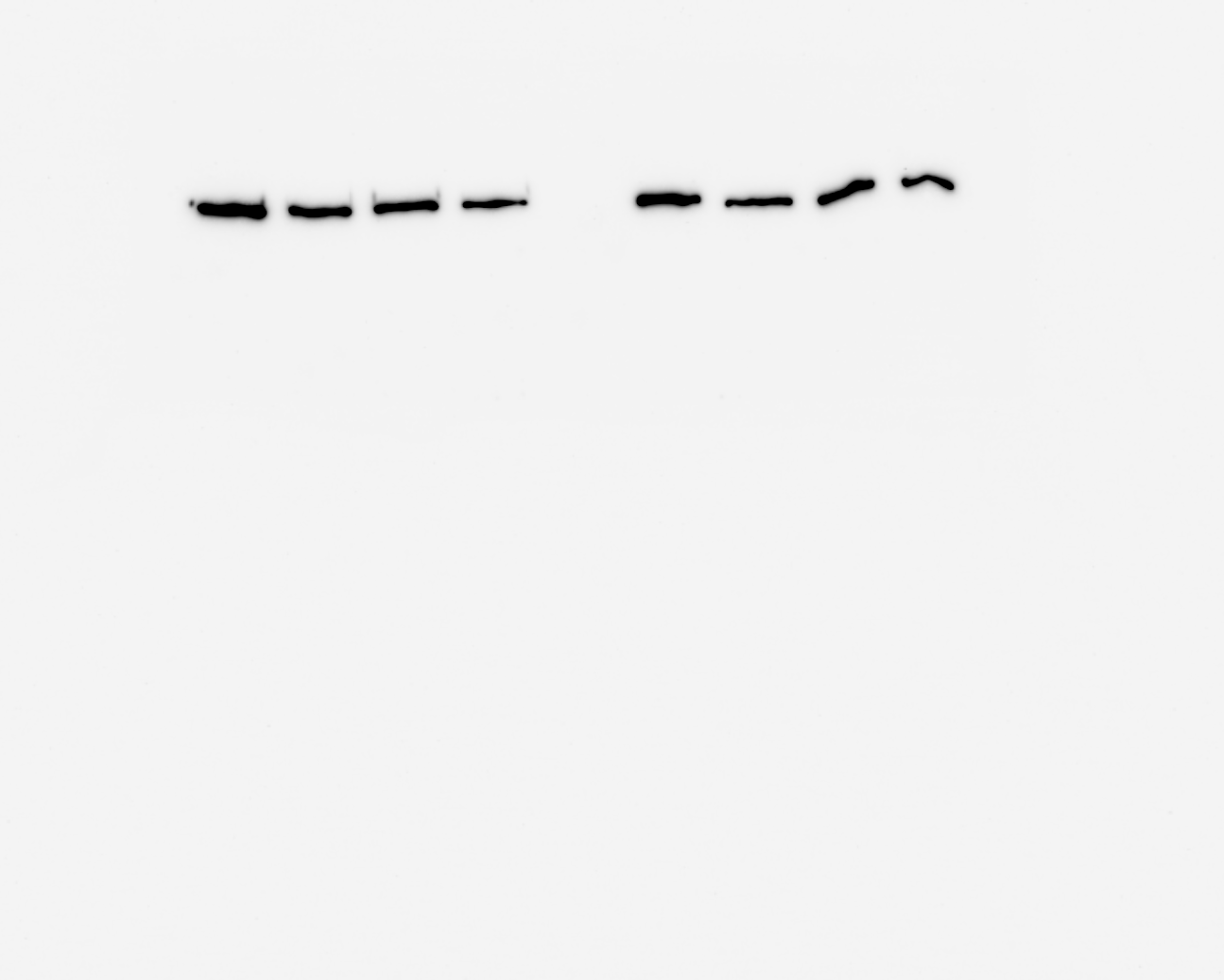

Supplement: Figure 5—source data 1. [file elife-108275-fig5-data1.zip › Figure 5-source data 1/Figure 5D_anti-Psm1.tif]

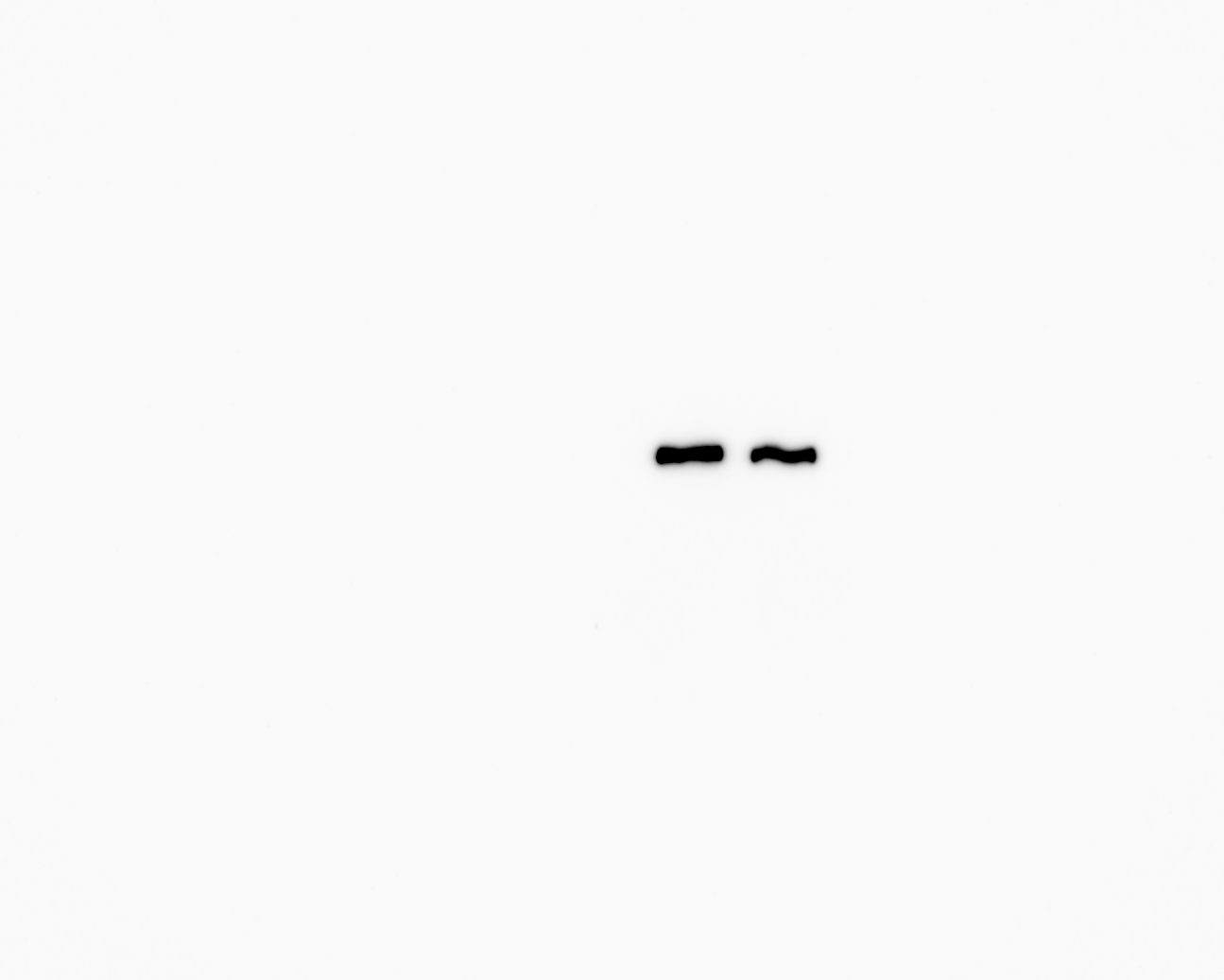

Supplement: Figure 5—source data 1. [file elife-108275-fig5-data1.zip › Figure 5-source data 1/Figure 5E_anti-GFP.tif]

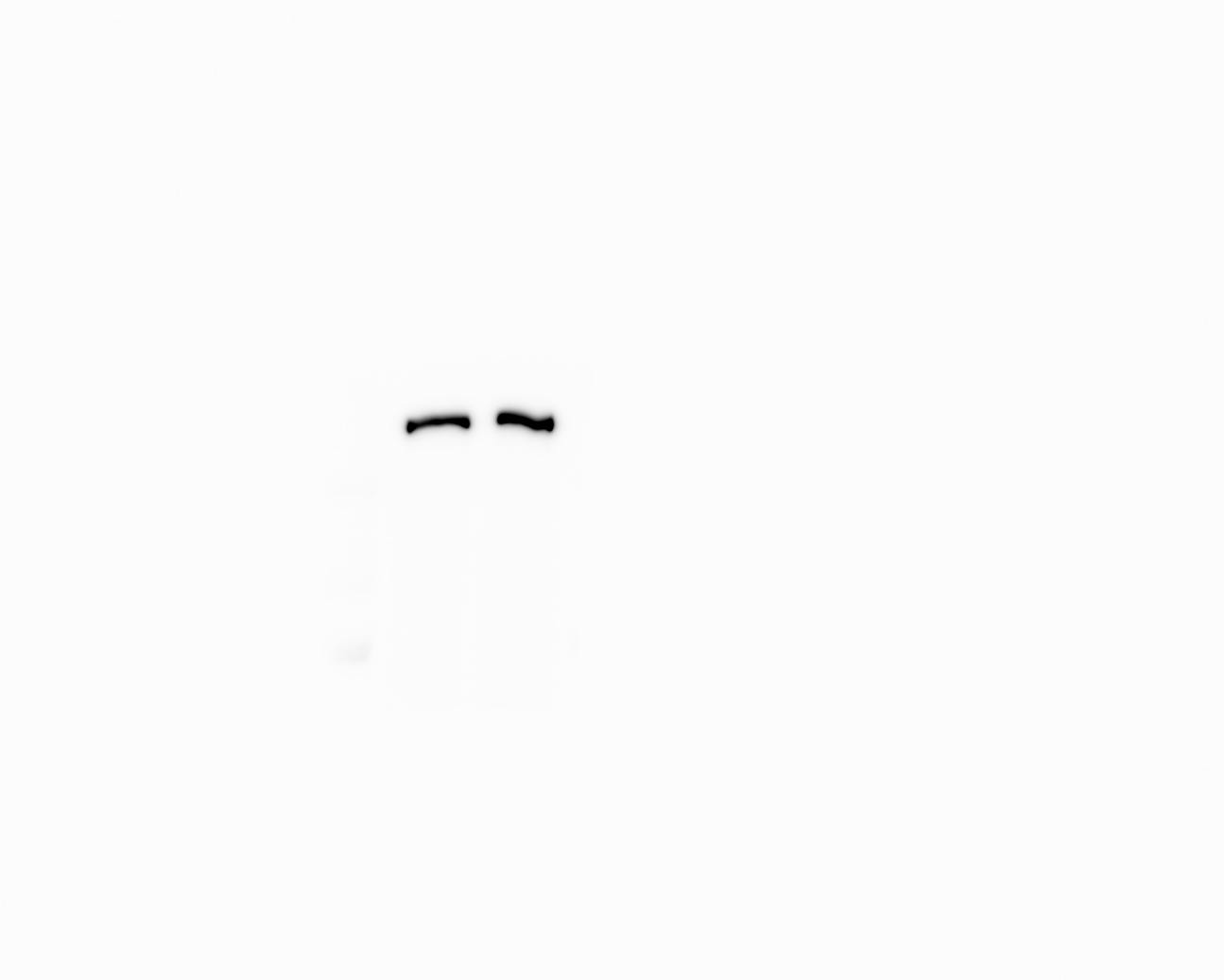

Supplement: Figure 5—source data 1. [file elife-108275-fig5-data1.zip › Figure 5-source data 1/Figure 5E_anti-Mis4-S183p.tif]

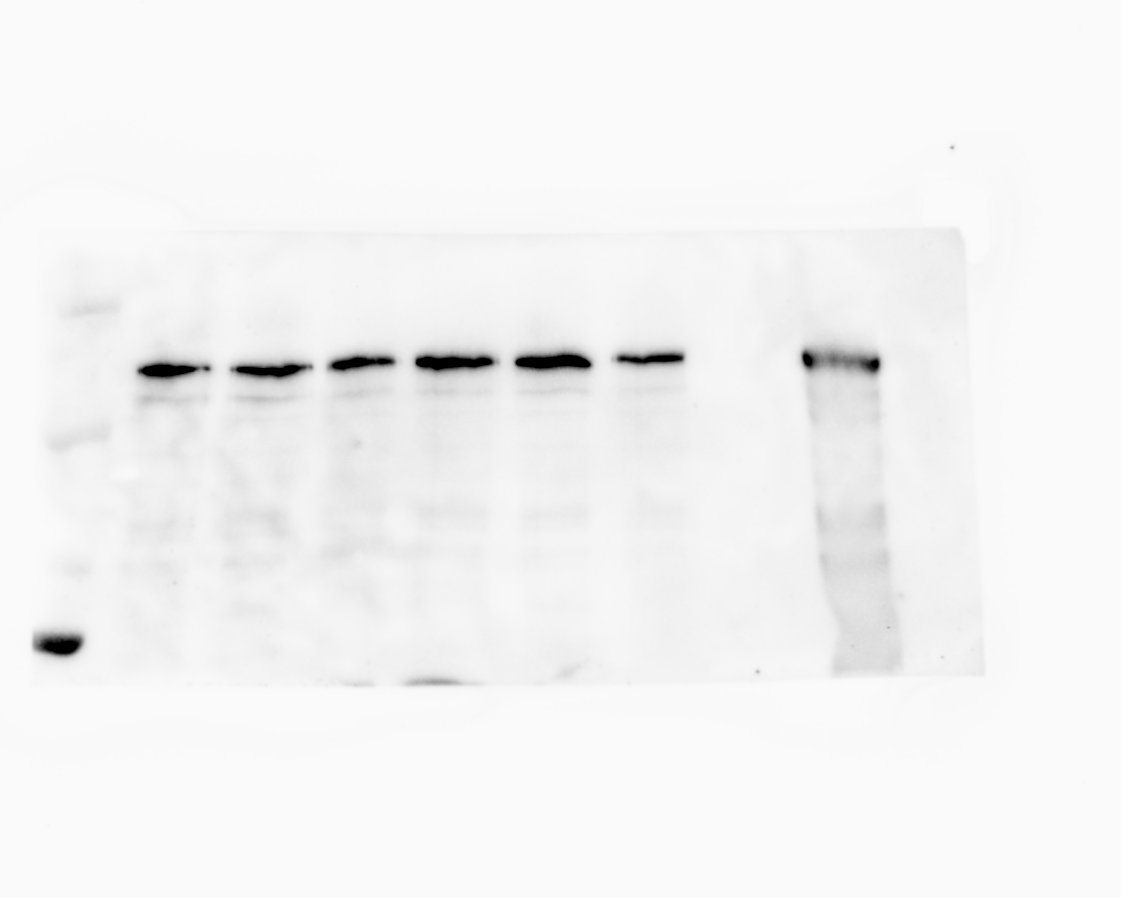

Supplement: Figure 5—figure supplement 1—source data 1. [file elife-108275-fig5-figsupp1-data1.zip › Figure 5-figure supplement 1-source data 1/Panel A_anti-GFP.tif]

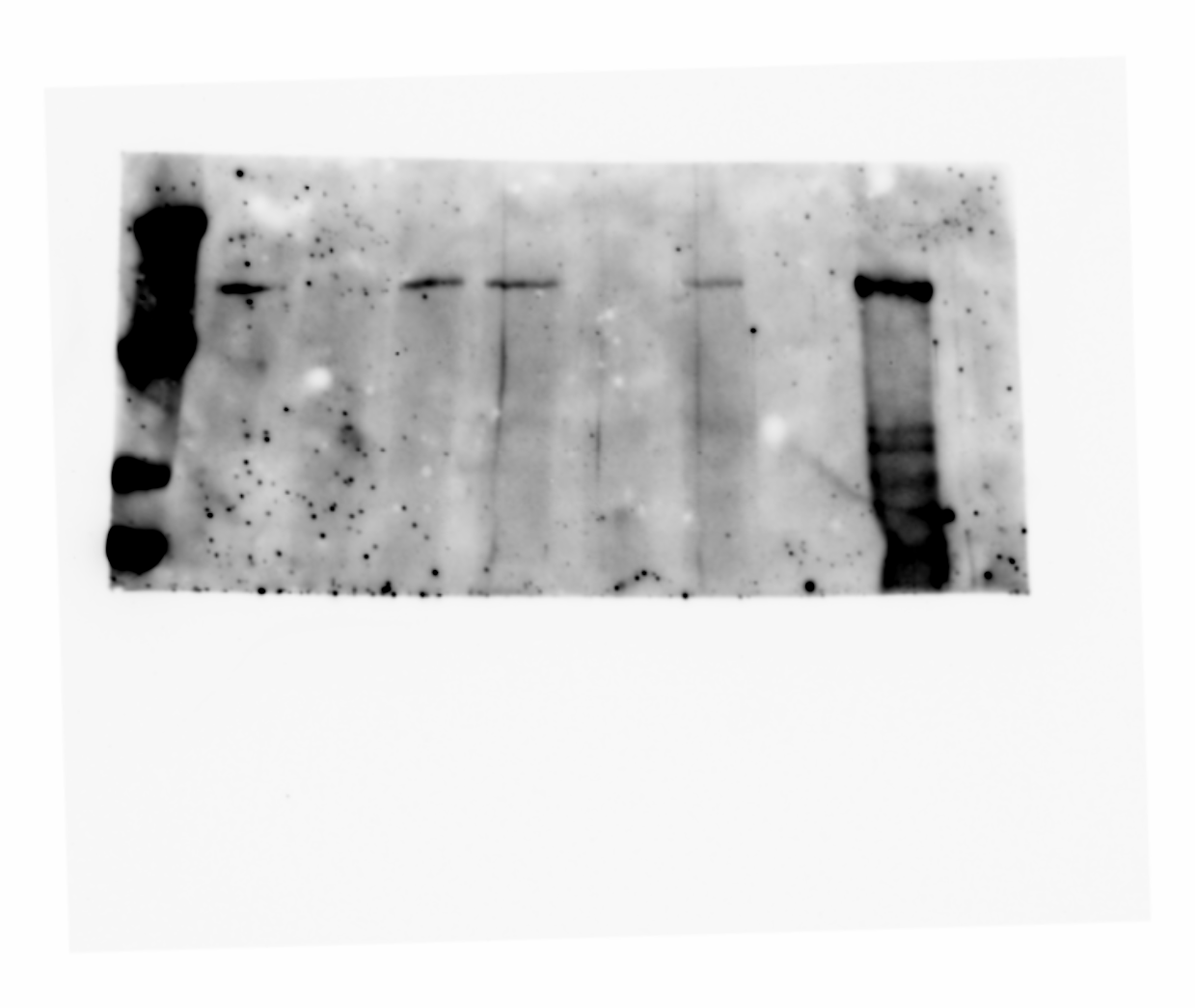

Supplement: Figure 5—figure supplement 1—source data 1. [file elife-108275-fig5-figsupp1-data1.zip › Figure 5-figure supplement 1-source data 1/Panel A_anti-Mis4-S183p.tif]

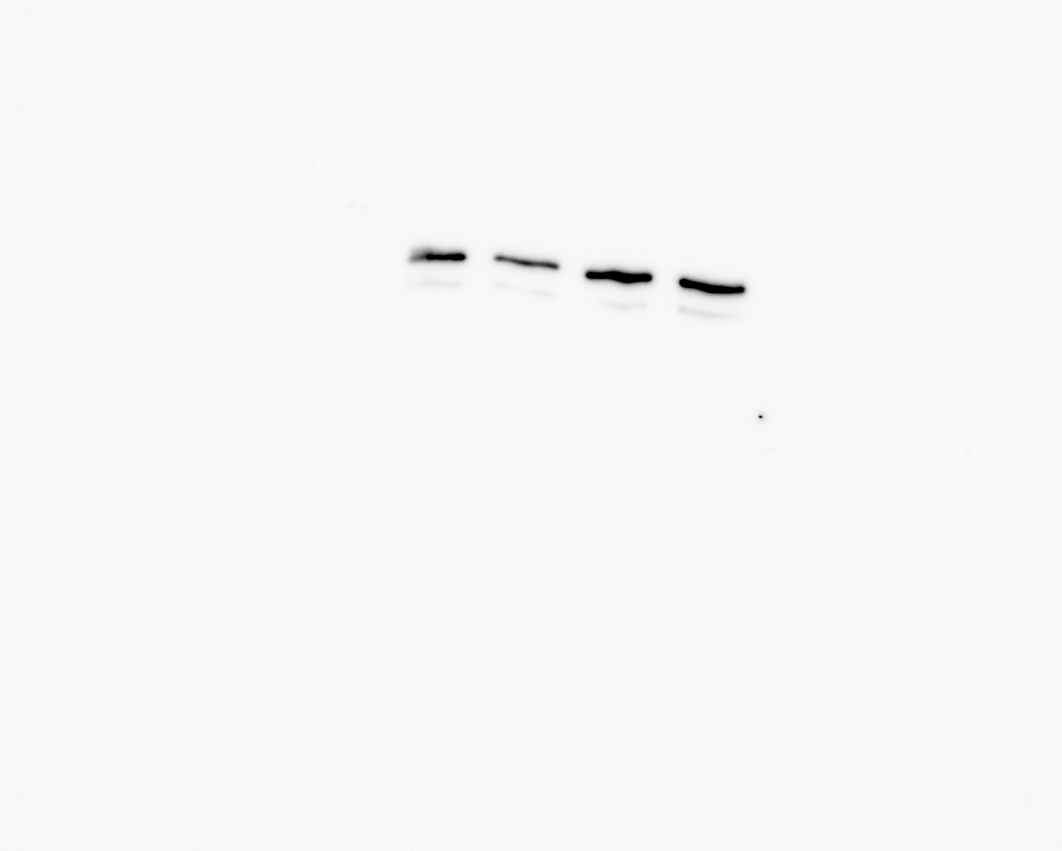

Supplement: Figure 5—figure supplement 1—source data 1. [file elife-108275-fig5-figsupp1-data1.zip › Figure 5-figure supplement 1-source data 1/Panel B_anti-GFP.tif]

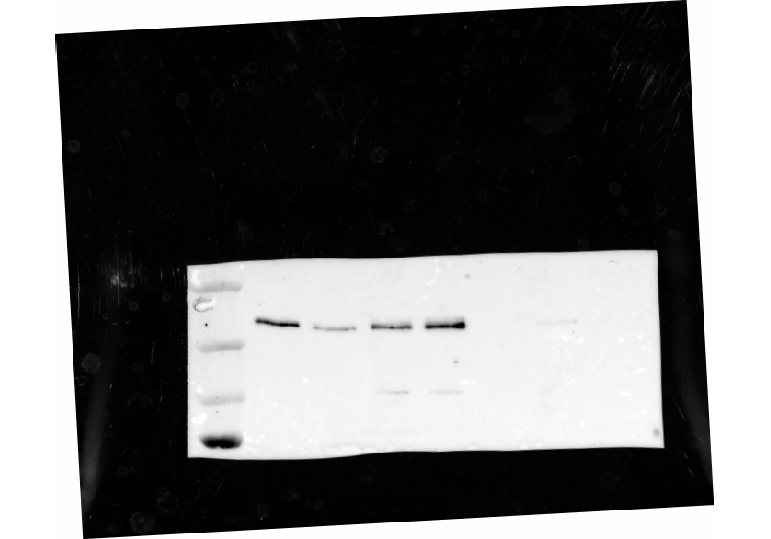

Supplement: Figure 5—figure supplement 1—source data 1. [file elife-108275-fig5-figsupp1-data1.zip › Figure 5-figure supplement 1-source data 1/Panel B_anti-Mis4-S183p.tif]

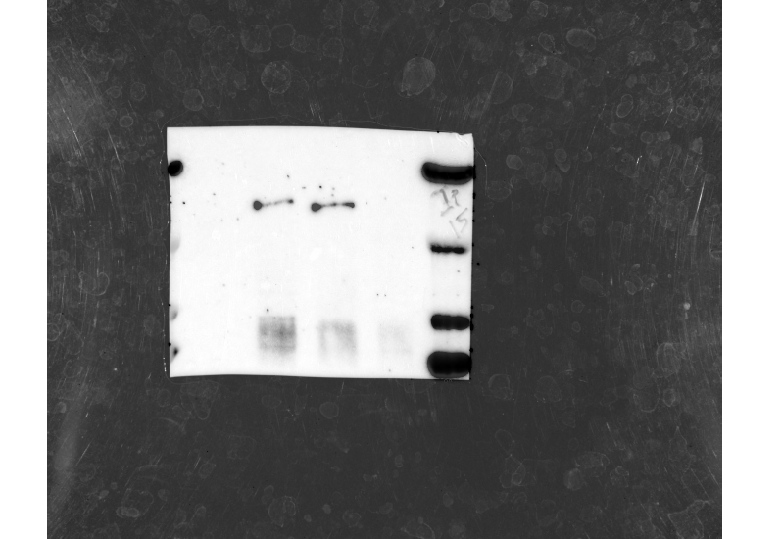

Supplement: Figure 5—figure supplement 2—source data 1. [file elife-108275-fig5-figsupp2-data1.zip › Figure 5-figure supplement 2-source data 1/Panel B_anti Mis4-S183p.tif]

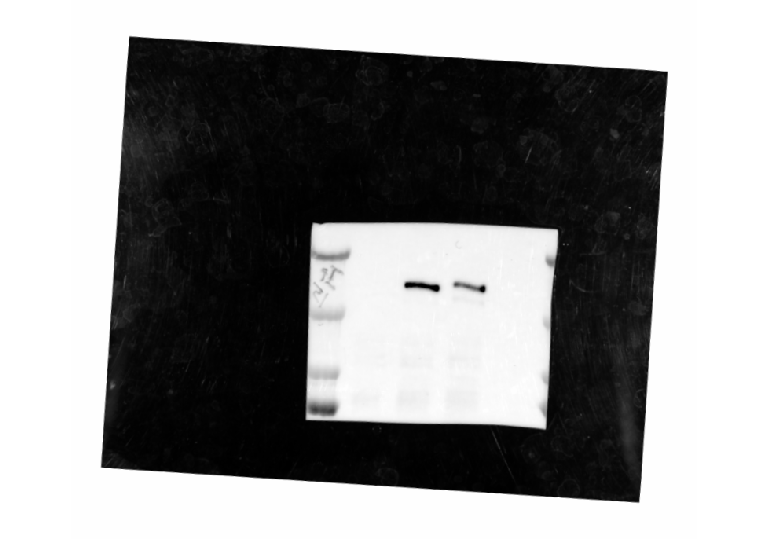

Supplement: Figure 5—figure supplement 2—source data 1. [file elife-108275-fig5-figsupp2-data1.zip › Figure 5-figure supplement 2-source data 1/Panel B_anti-GFP.tif]

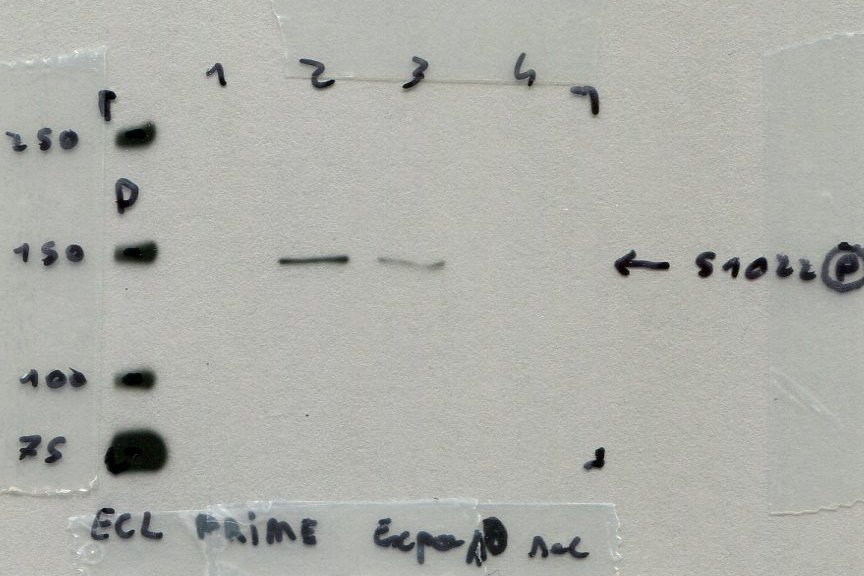

Supplement: Figure 5—figure supplement 2—source data 1. [file elife-108275-fig5-figsupp2-data1.zip › Figure 5-figure supplement 2-source data 1/Panel D Left_anti-Psm1-S1022p.jpg]

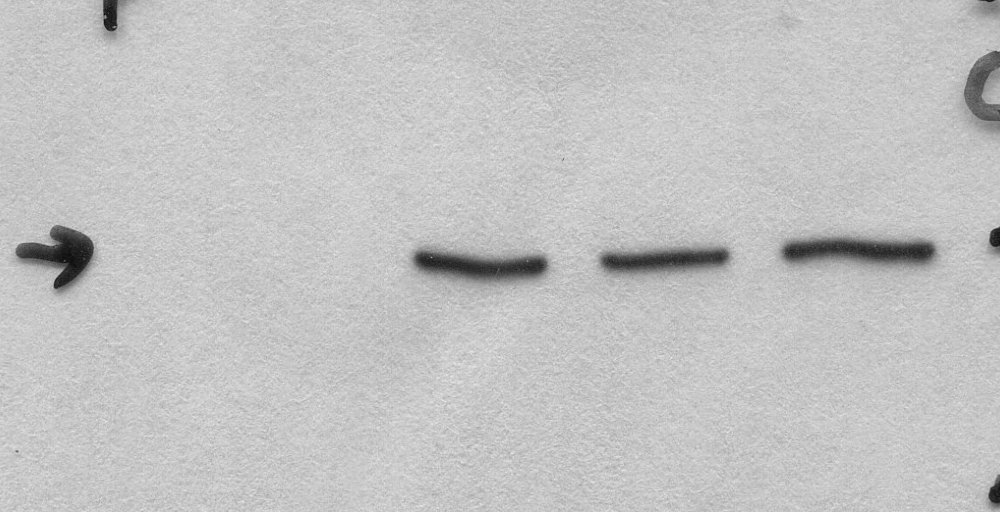

Supplement: Figure 5—figure supplement 2—source data 1. [file elife-108275-fig5-figsupp2-data1.zip › Figure 5-figure supplement 2-source data 1/Panel D Left_anti-Psm1.jpg]

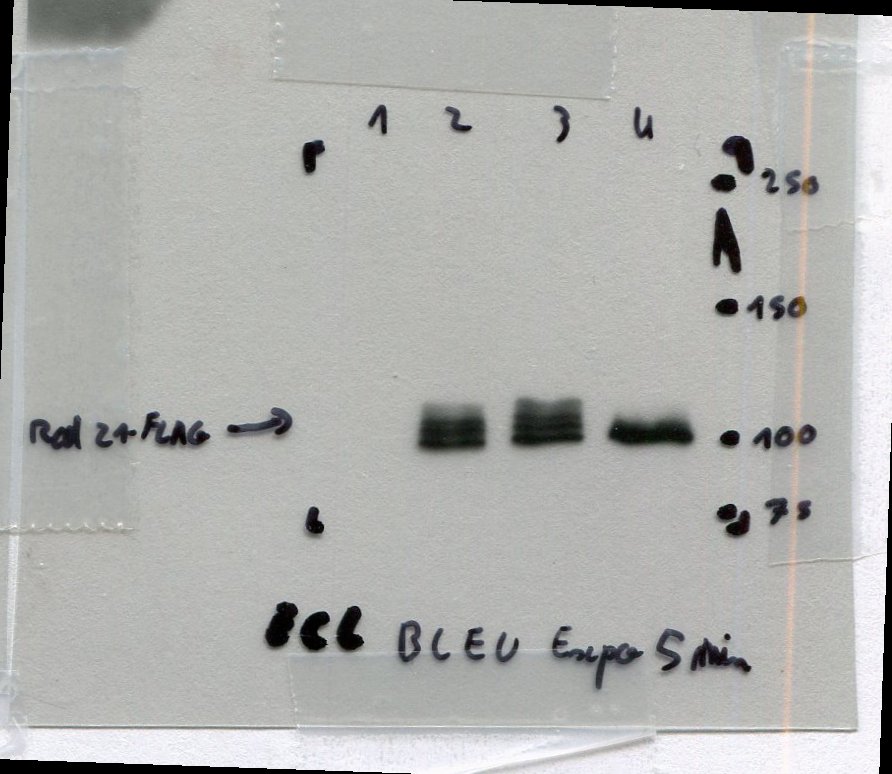

Supplement: Figure 5—figure supplement 2—source data 1. [file elife-108275-fig5-figsupp2-data1.zip › Figure 5-figure supplement 2-source data 1/Panel D Right_anti-FLAG.jpg]

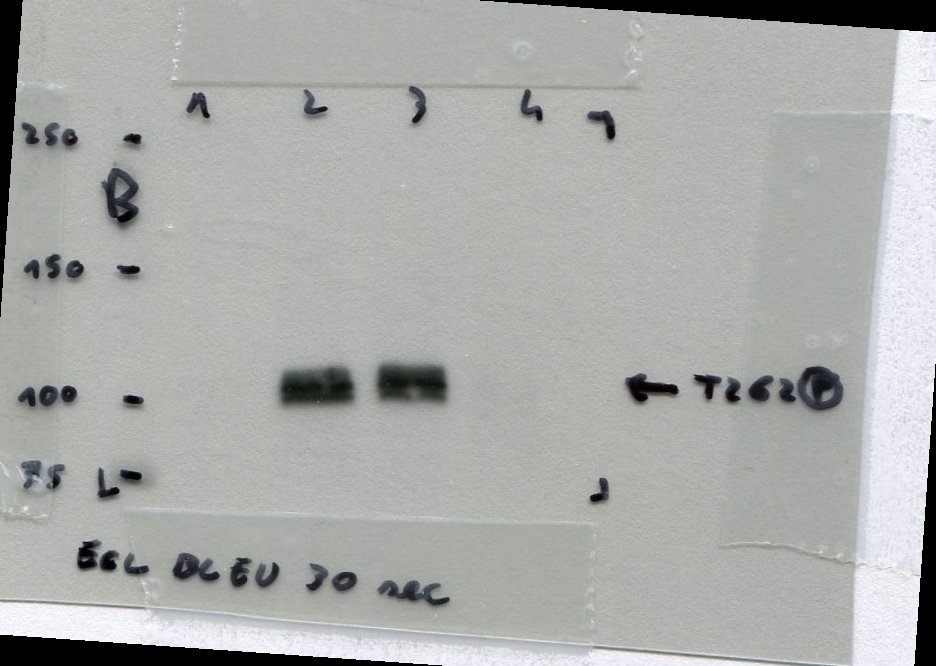

Supplement: Figure 5—figure supplement 2—source data 1. [file elife-108275-fig5-figsupp2-data1.zip › Figure 5-figure supplement 2-source data 1/Panel D Right_anti-Rad21-T262p.jpg]

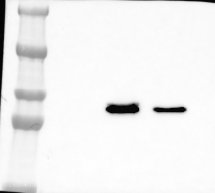

Supplement: Figure 5—figure supplement 2—source data 1. [file elife-108275-fig5-figsupp2-data1.zip › Figure 5-figure supplement 2-source data 1/Panel E_anti-myc.jpg]

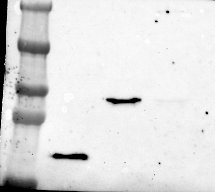

Supplement: Figure 5—figure supplement 2—source data 1. [file elife-108275-fig5-figsupp2-data1.zip › Figure 5-figure supplement 2-source data 1/Panel E_anti-Psk1p.jpg]

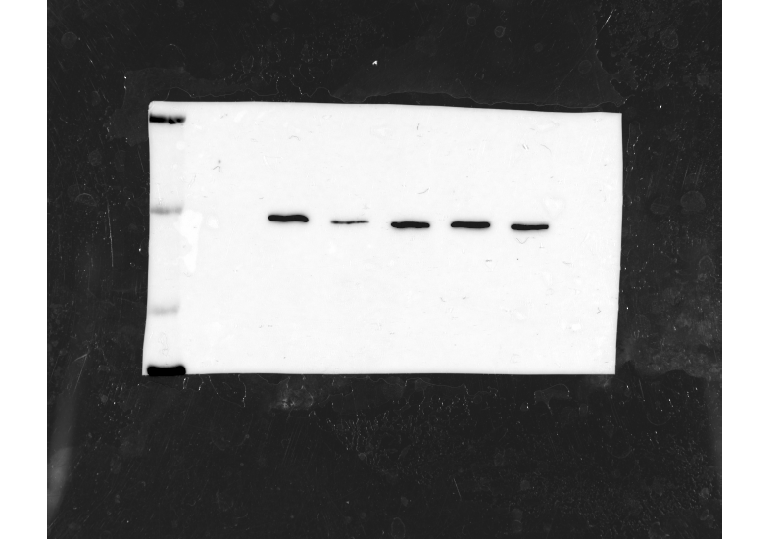

Supplement: Figure 5—figure supplement 3—source data 1. [file elife-108275-fig5-figsupp3-data1.zip › Figure 5-figure supplement 3-source data 1/Panel D_anti-Psm1-S1022p.tif]

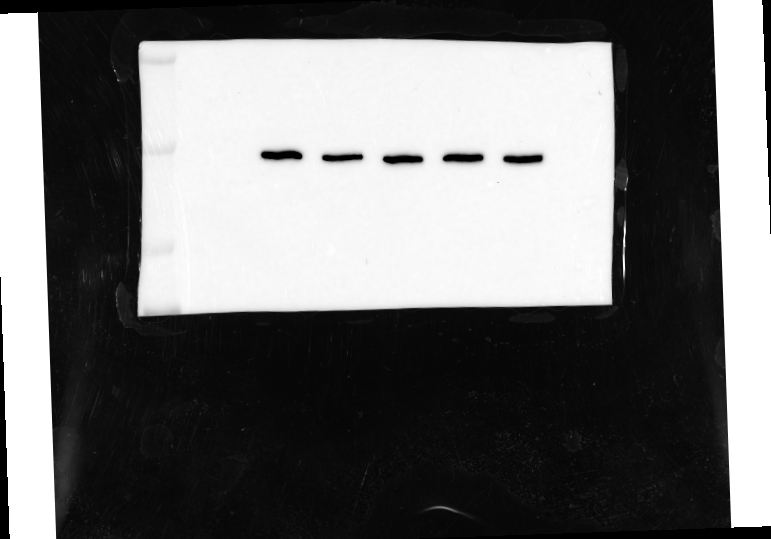

Supplement: Figure 5—figure supplement 3—source data 1. [file elife-108275-fig5-figsupp3-data1.zip › Figure 5-figure supplement 3-source data 1/Panel D_anti-Psm1.tif]

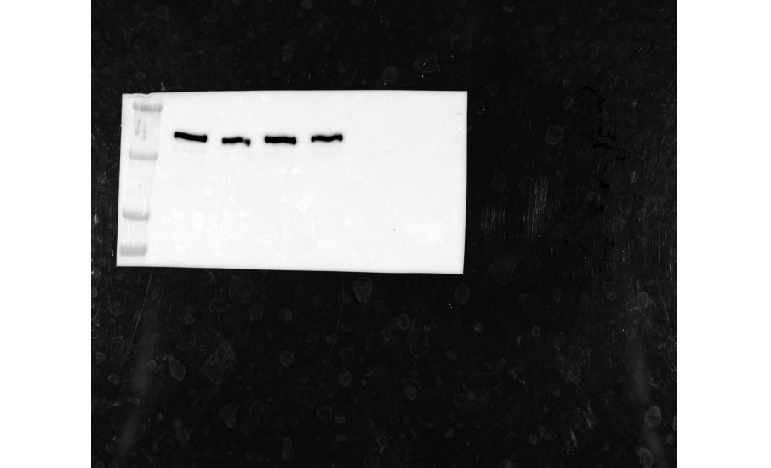

Supplement: Figure 5—figure supplement 3—source data 1. [file elife-108275-fig5-figsupp3-data1.zip › Figure 5-figure supplement 3-source data 1/Panel E_anti-GFP.tif]

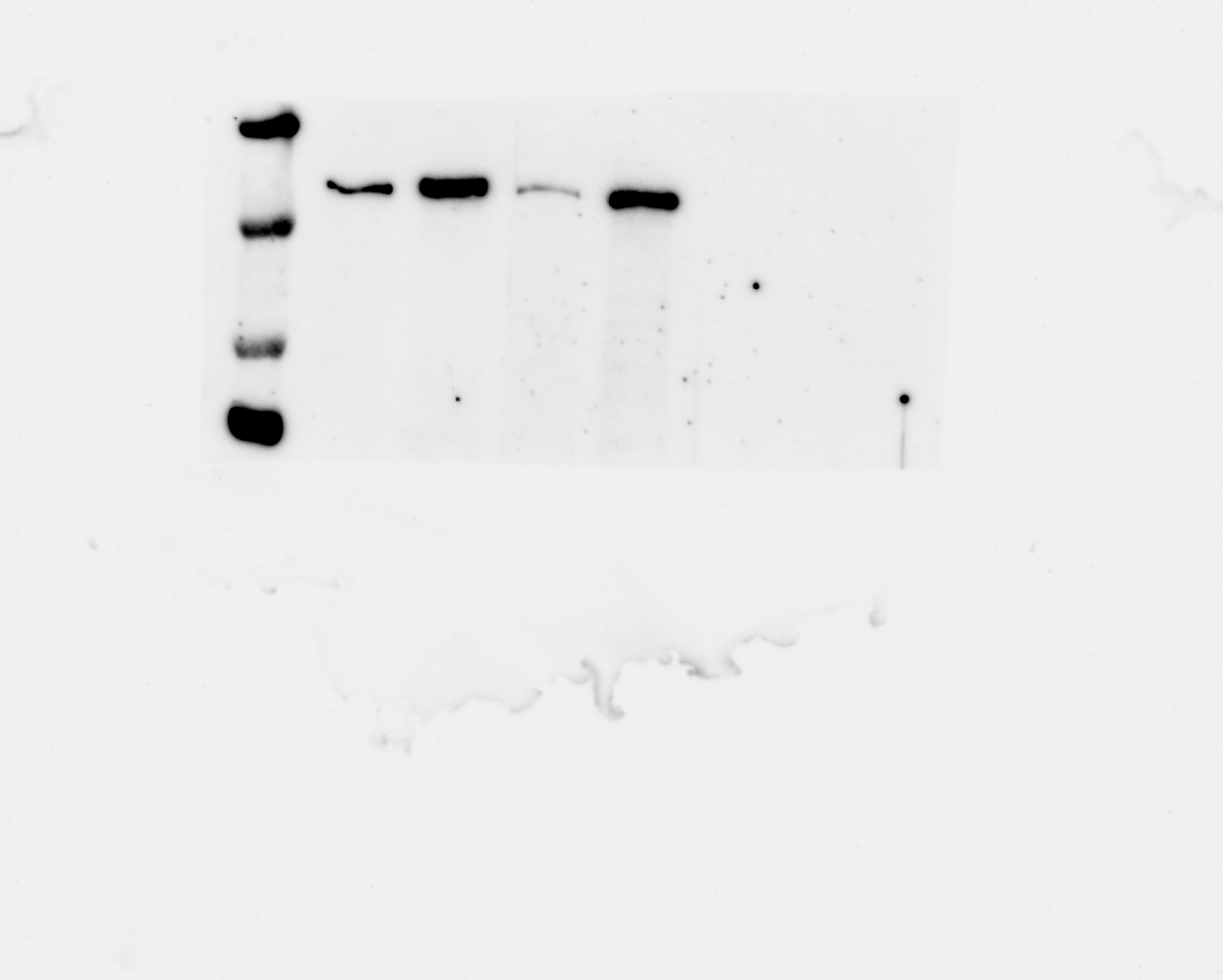

Supplement: Figure 5—figure supplement 3—source data 1. [file elife-108275-fig5-figsupp3-data1.zip › Figure 5-figure supplement 3-source data 1/Panel E_anti-Mis4-S183p.tif]
