# Supplementary material for: Interplay between cohesin and TORC1 links chromosome segregation and gene expression to environmental changes
Source: eLife. 2026 Jun 1;14:RP108275. doi: 10.7554/eLife.108275 (PMC13225845; doi:10.7554/eLife.108275)

Figure 1-source data 2. Composite of Figure 1C. The original images are on the left; the final composite is on the right.

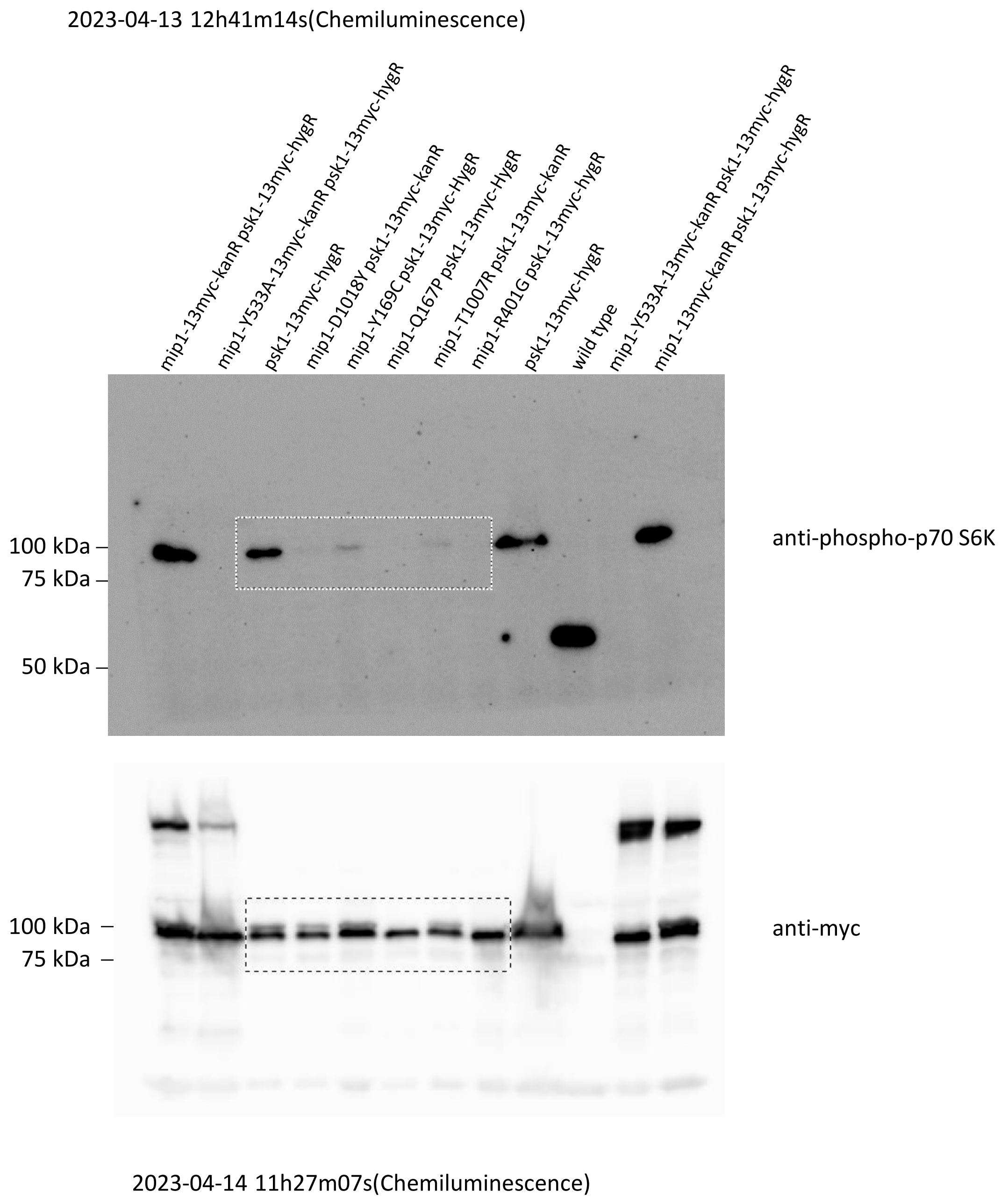

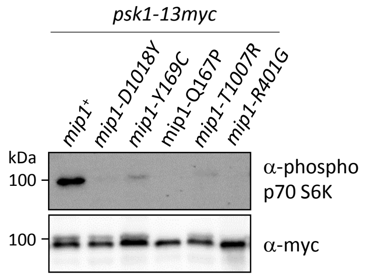

Supplement: Figure 1—source data 2. [file elife-108275-fig1-data2.zip › Figure 1-source data 2/Figure 1–source data 2.pdf]
