# Supplementary material for: Interplay between cohesin and TORC1 links chromosome segregation and gene expression to environmental changes
Source: eLife. 2026 Jun 1;14:RP108275. doi: 10.7554/eLife.108275 (PMC13225845; doi:10.7554/eLife.108275)

Figure 2-figure supplement 3-source data 2. Composite for panel B. The original images are at the top; the final composite is at the bottom.

Membrane 1: anti-Psm3-K106Ac

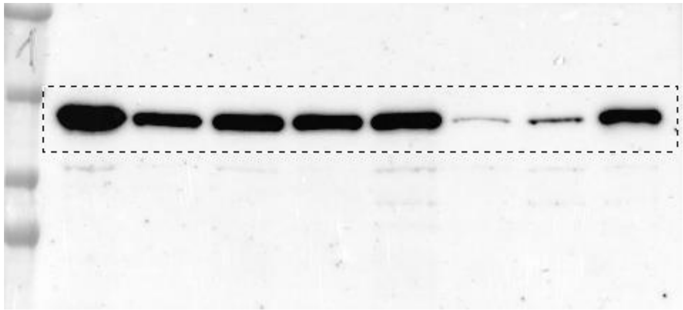

Membrane 2: anti-Psm3

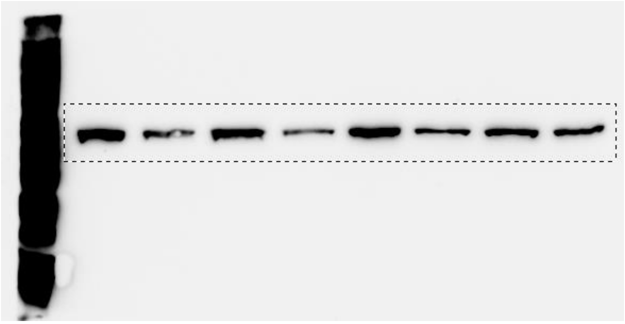

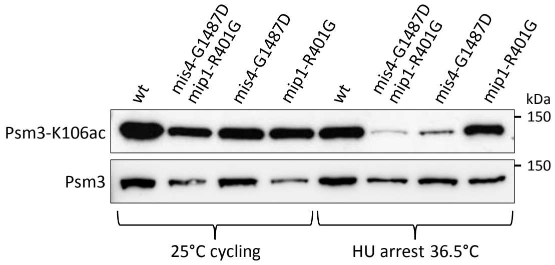

Supplement: Figure 2—figure supplement 3—source data 2. [file elife-108275-fig2-figsupp3-data2.zip › Figure 2-figure supplement 3-source data 2/Figure 2–figure supplement 3-source data 2.pdf]
