# Supplementary material for: Interplay between cohesin and TORC1 links chromosome segregation and gene expression to environmental changes
Source: eLife. 2026 Jun 1;14:RP108275. doi: 10.7554/eLife.108275 (PMC13225845; doi:10.7554/eLife.108275)

Figure 4-source data 2. The original images are at the top; the final composite is at the bottom.

Figure 4B

INPUT

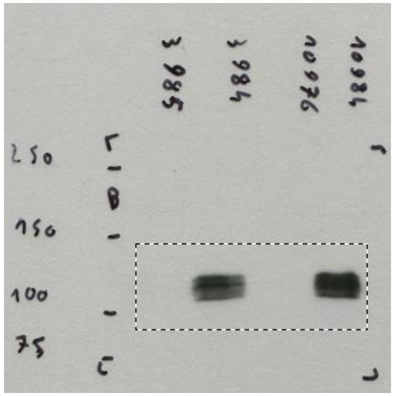

Figure 4B_INPUT_blot PK

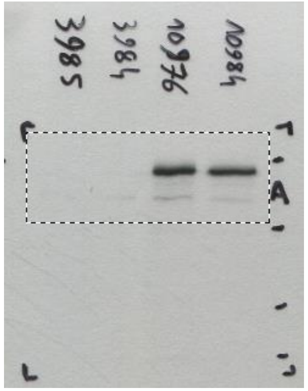

Figure 4B_INPUT_blot FLAG

IP anti-PK

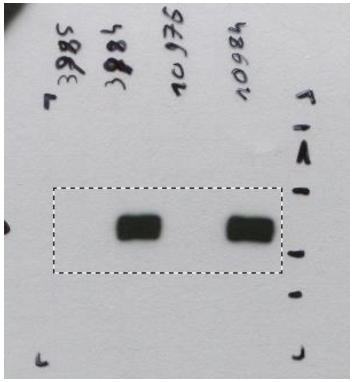

Figure 4B_IP PK_blot PK

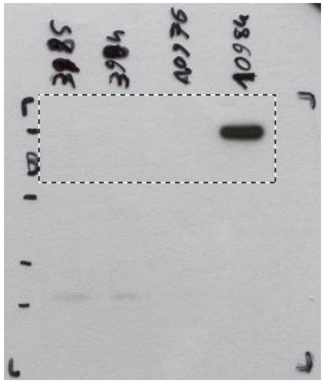

Figure 4B_IP PK_blot FLAG

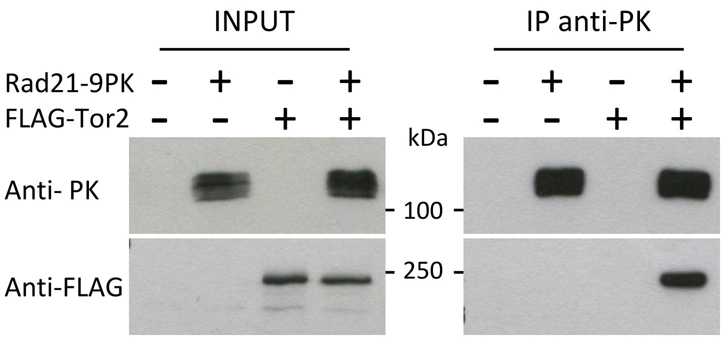

Figure 4C-left

INPUT | IP anti-FLAG

Anti-FLAG

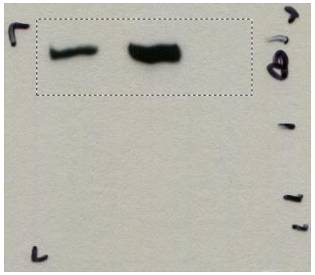

Figure 4C-left-INPUT-blot FLAG

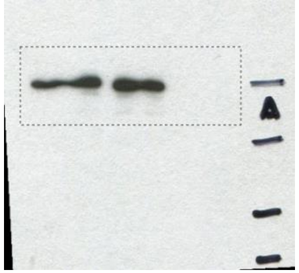

Figure 4C-left-IP FLAG-blot FLAG

Anti-GFP

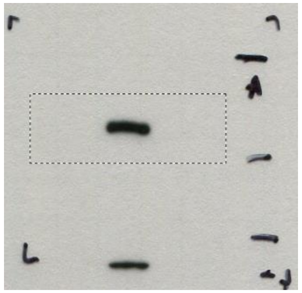

Figure 4C-left-INPUT-blot GFP

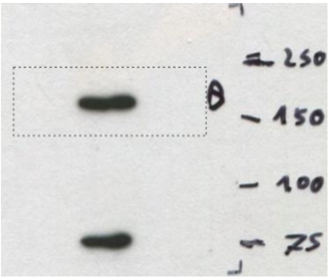

Figure 4C-left-IP FLAG-blot GFP

Anti-Rad21

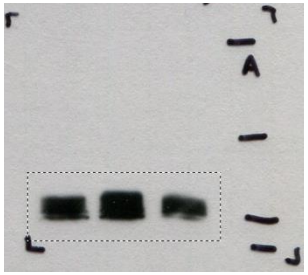

Figure 4C-left-INPUT-blot Rad21

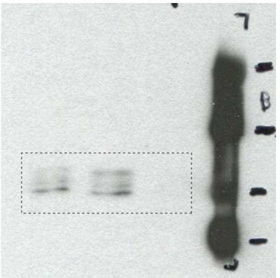

Figure 4C-left-IP FLAG-blot Rad21

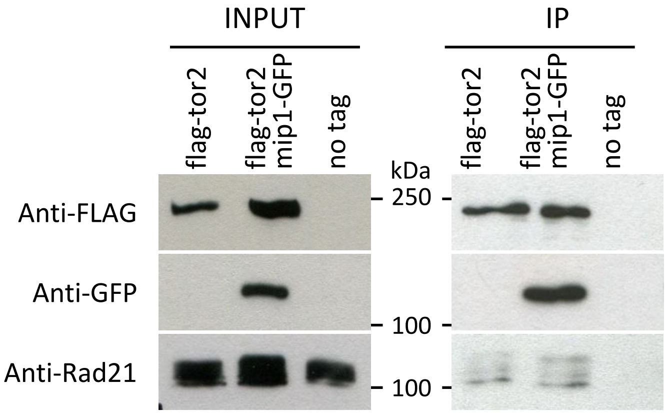

Figure 4C-right

INPUT

IP

Anti-FLAG

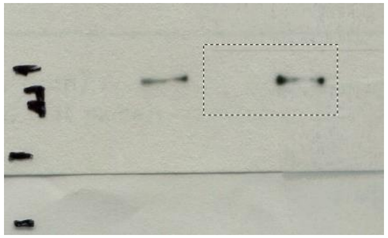

Figure 4C-right-INPUT-blot Psm1&FLAG

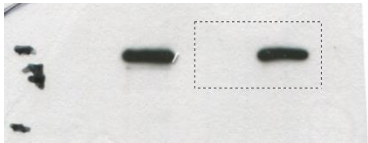

Figure 4C-right-IP FLAG-blot FLAG

Anti-GFP

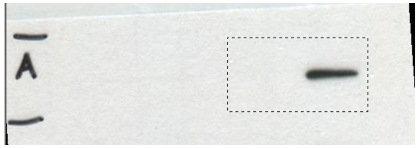

Figure 4C-right-INPUT-blot GFP&Rad21

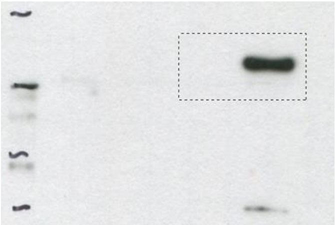

Figure 4C-right-IP-blot GFP

Anti-Psm1

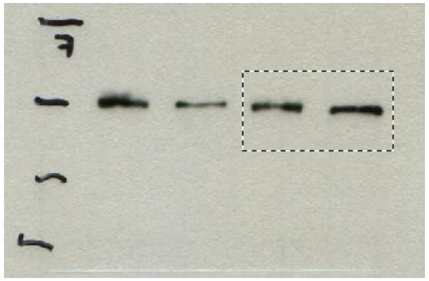

Figure 4C-right-INPUT-blot Psm1&FLAG

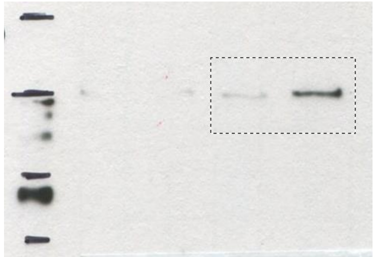

Figure 4C-right-IP-blot Psm1

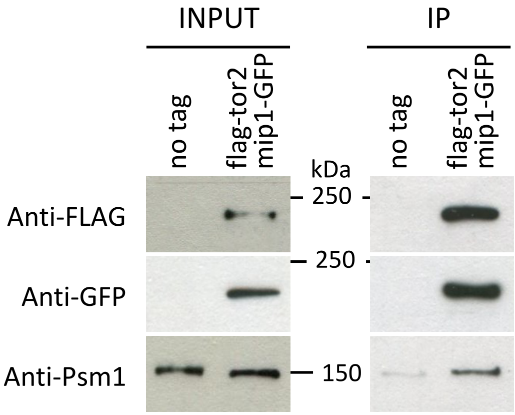

Supplement: Figure 4—source data 2. [file elife-108275-fig4-data2.zip › Figure 4-source data 2/Figure 4–source data 2.pdf]
