# Supplementary material for: Interplay between cohesin and TORC1 links chromosome segregation and gene expression to environmental changes
Source: eLife. 2026 Jun 1;14:RP108275. doi: 10.7554/eLife.108275 (PMC13225845; doi:10.7554/eLife.108275)
Supplement: Figure 4—figure supplement 1—source data 2. [file elife-108275-fig4-figsupp1-data2.zip › Figure 4-figure supplement 1-source data 2/Figure 4-figure supplement 1-source data 2.pdf]

Figure 4-figure supplement 1-source data 2. Composite for panel B and C. The original images are at the top; the final composite is at the bottom.

Panel B

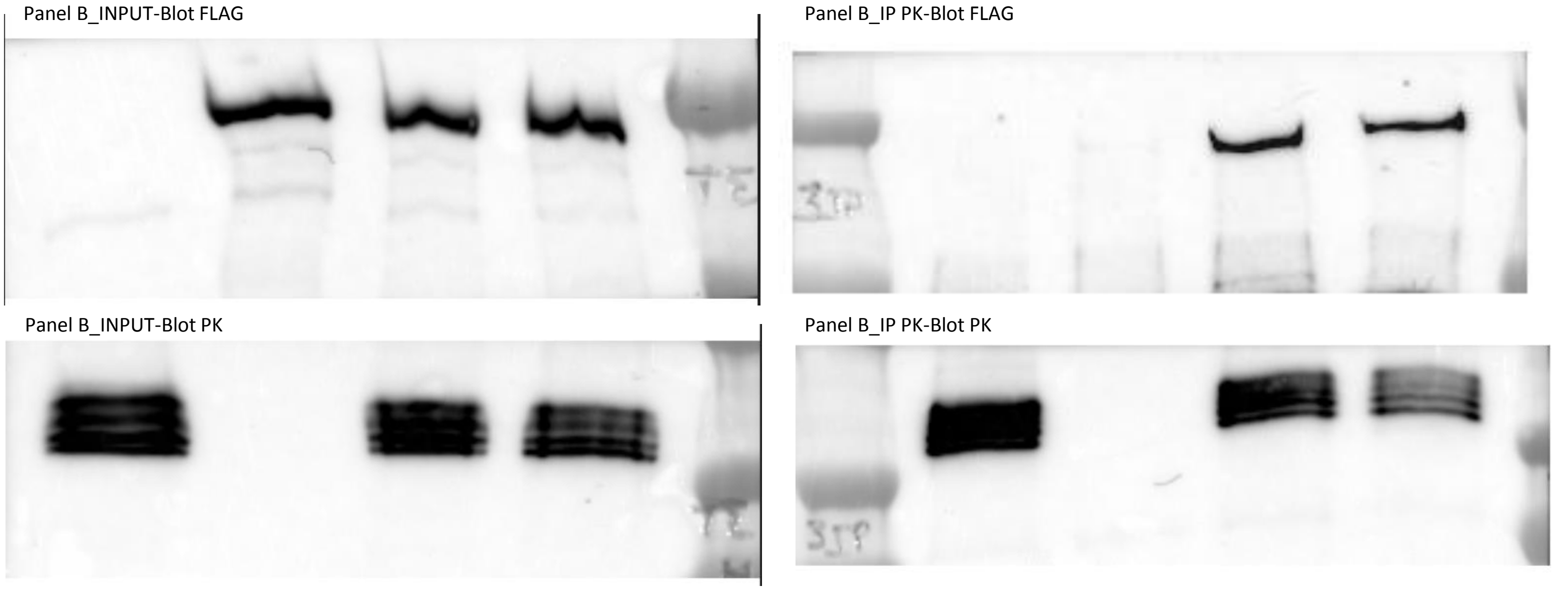

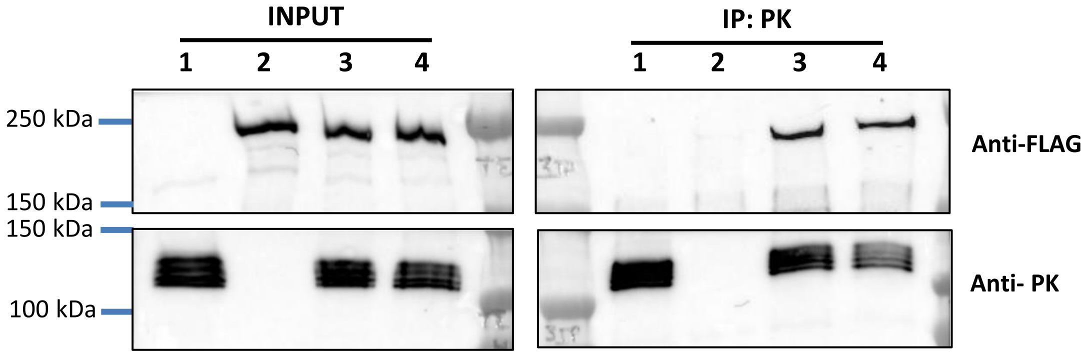

Panel C

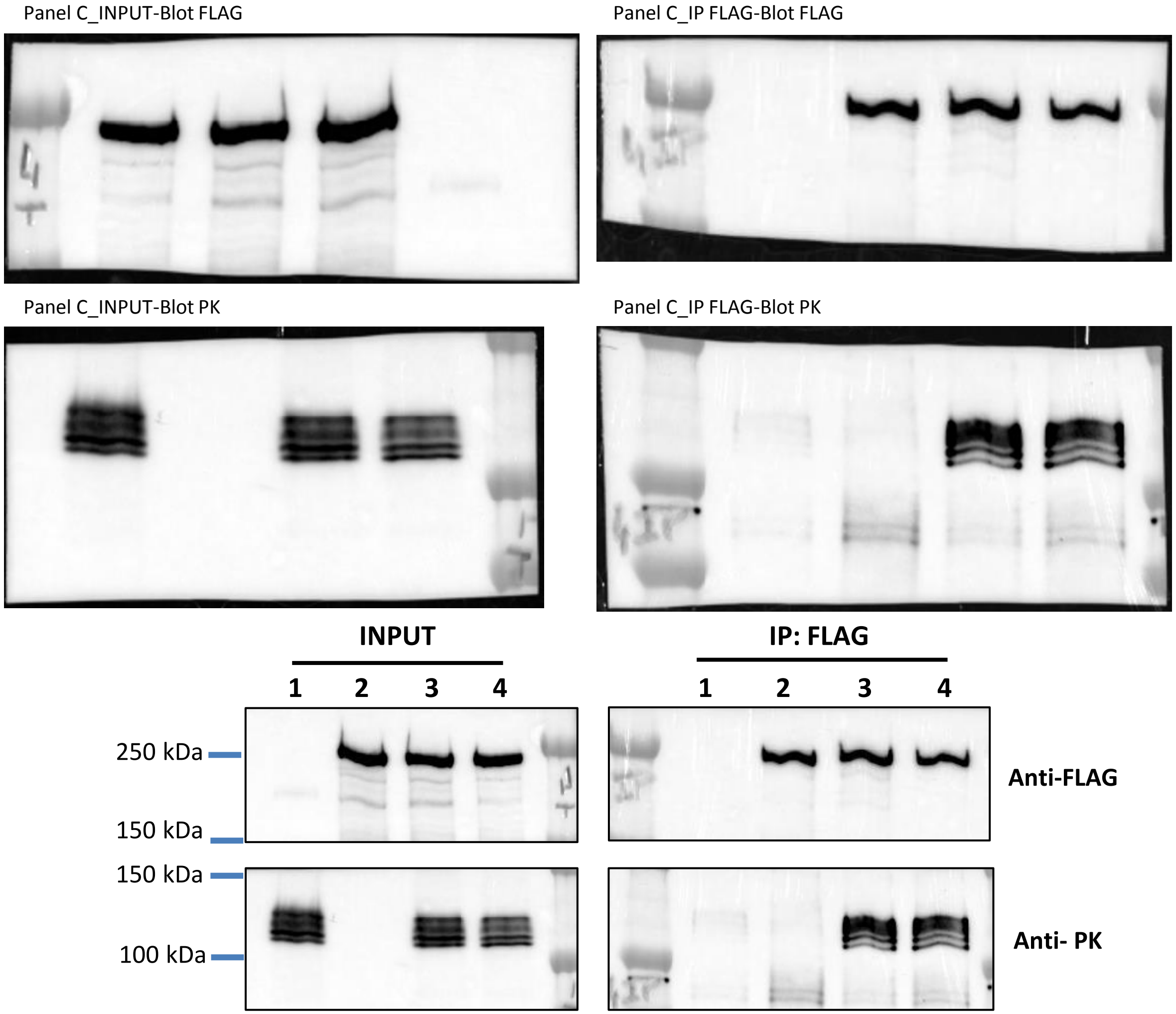
Panel C_INPUT-Blot FLAG
Panel C_IP FLAG-Blot FLAG
Panel C_INPUT-Blot PK
Panel C_IP FLAG-Blot PK
INPUT
IP: FLAG
1 2 3 4
1 2 3 4
250 kDa
150 kDa
150 kDa
100 kDa
Anti-FLAG
Anti- PK
