# Supplementary material for: Interplay between cohesin and TORC1 links chromosome segregation and gene expression to environmental changes
Source: eLife. 2026 Jun 1;14:RP108275. doi: 10.7554/eLife.108275 (PMC13225845; doi:10.7554/eLife.108275)

Figure 5-source data 2. The original images are on the left; the final composite is on the right.

Figure 5B

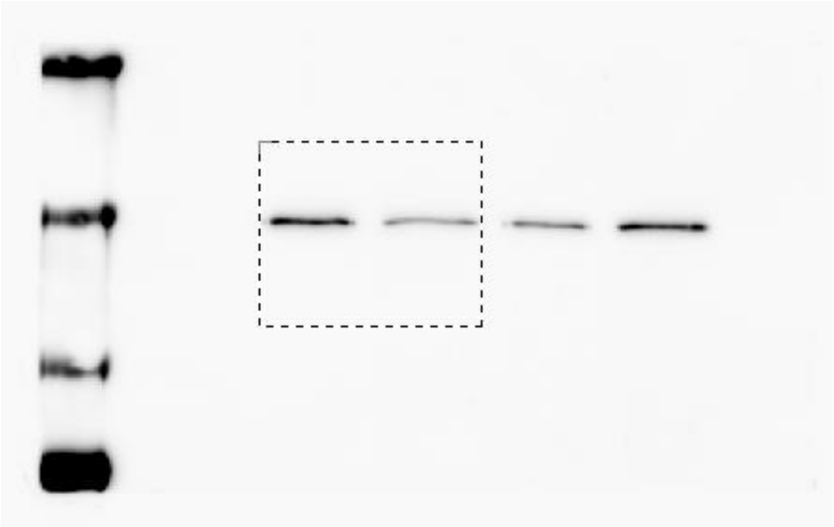

Figure 5B_anti-Psm1-S1022p

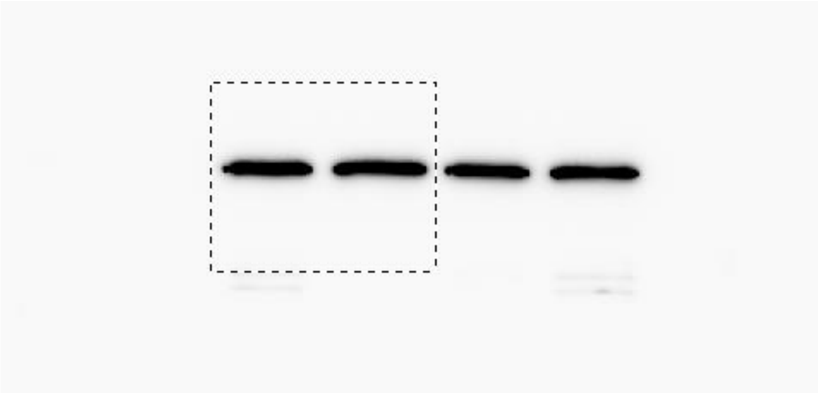

Figure 5B_anti-Psm1

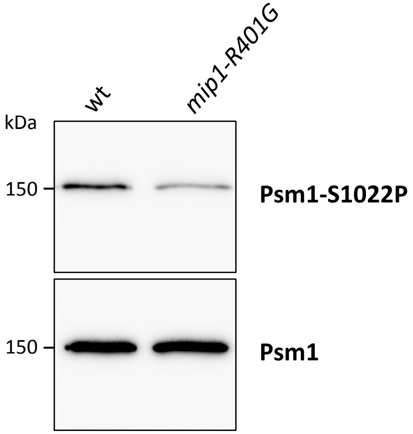

Figure 5C

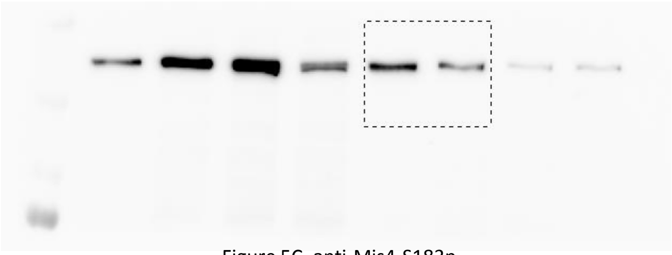

Figure 5C_anti-Mis4-S183p

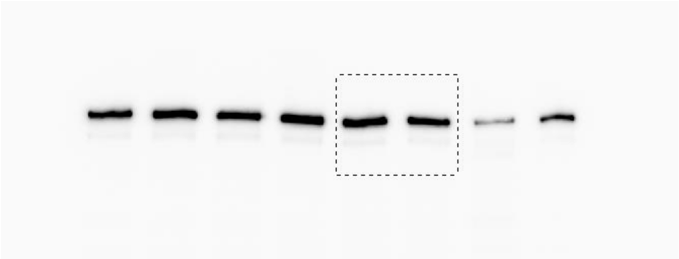

Figure 5C_anti-GFP

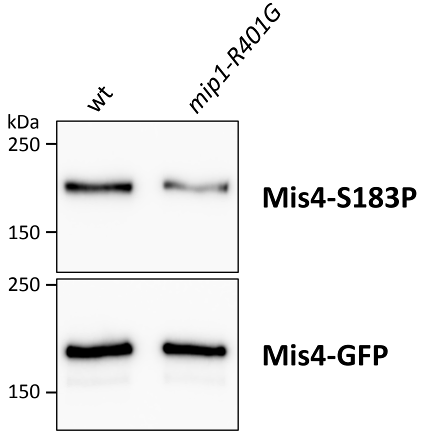

Figure 5D

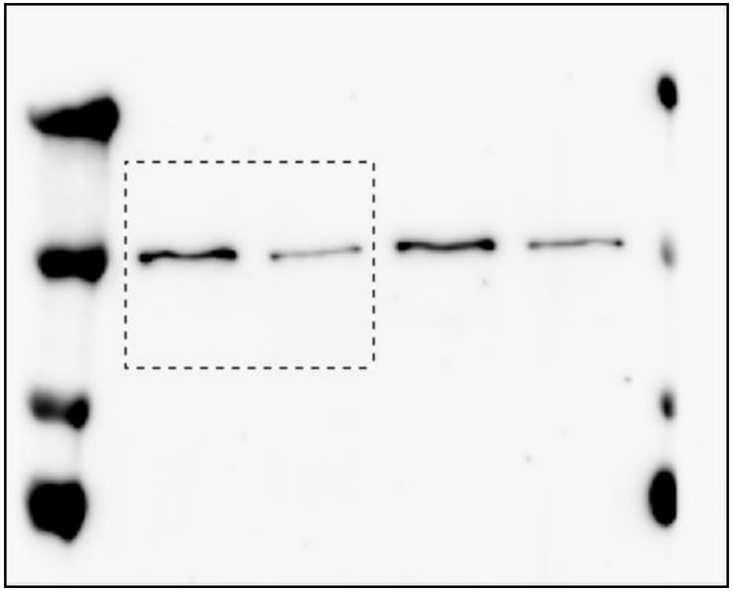

Figure 5D_anti-Psm1-S1022p

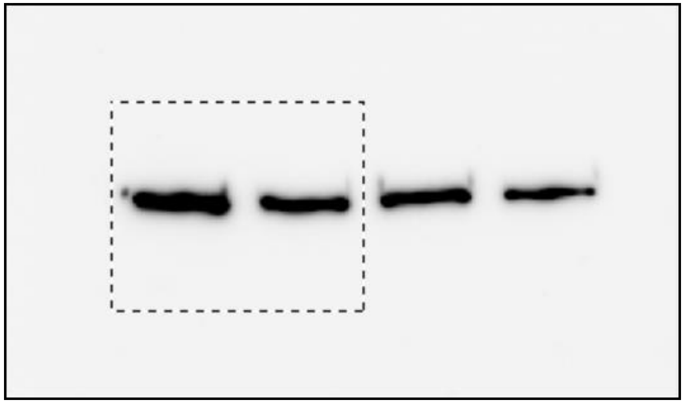

Figure 5D_anti-Psm1

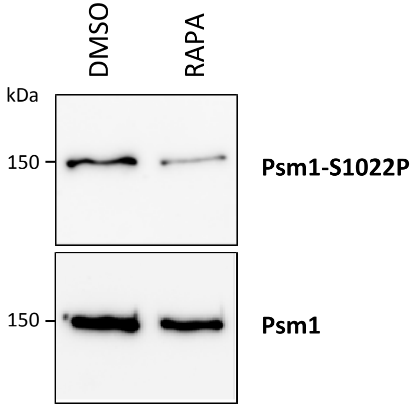

Figure 5E

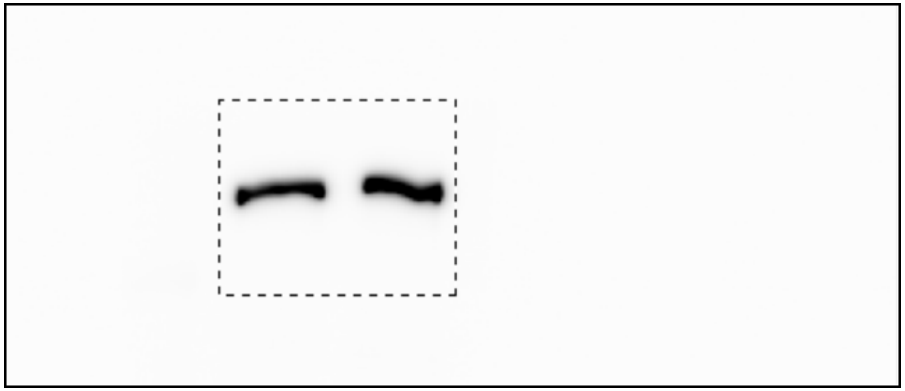

Figure 5E_anti-Mis4-S183p

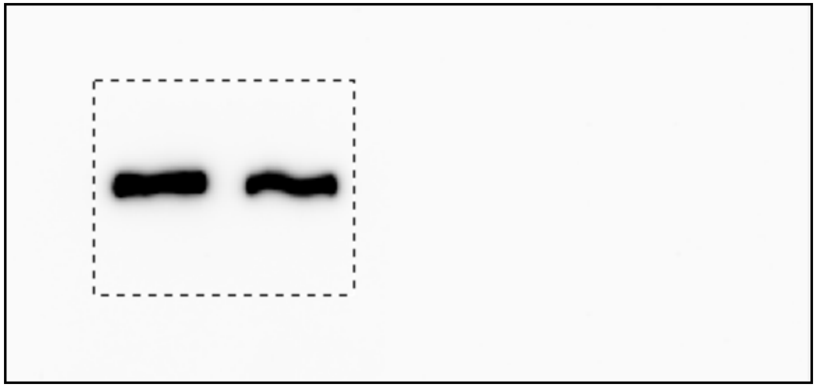

Figure 5E_anti-GFP

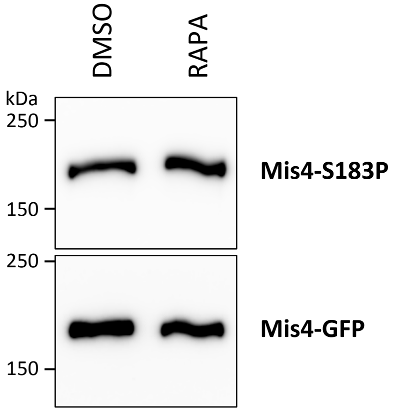

Supplement: Figure 5—source data 2. [file elife-108275-fig5-data2.zip › Figure 5-source data 2/Figure 5–source data 2.pdf]
