# Supplementary material for: Interplay between cohesin and TORC1 links chromosome segregation and gene expression to environmental changes
Source: eLife. 2026 Jun 1;14:RP108275. doi: 10.7554/eLife.108275 (PMC13225845; doi:10.7554/eLife.108275)

Figure 5-figure supplement 1-source data 2. The original images are on the left; the final composite is on the right.

Panel A

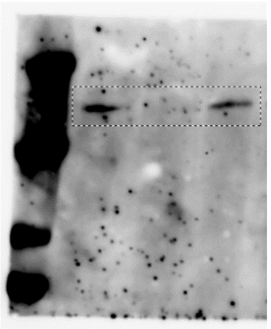

Panel A_anti-Mis4-S183p

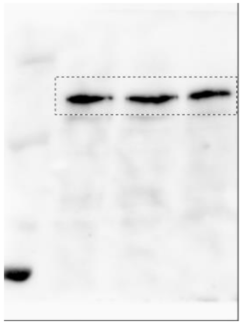

Panel A_anti-GFP

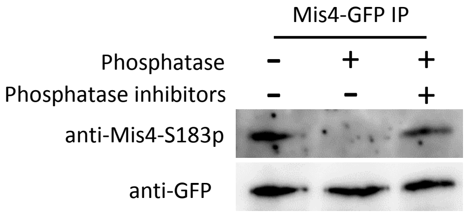

Panel B

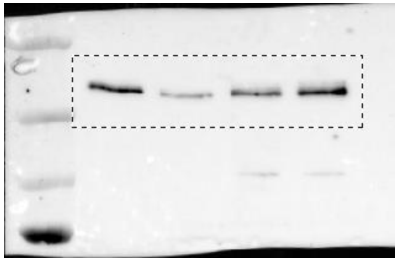

Panel B_anti-Mis4-S183p

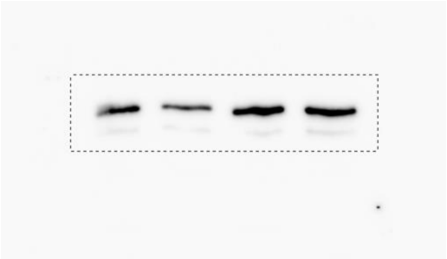

Panel B_anti-GFP

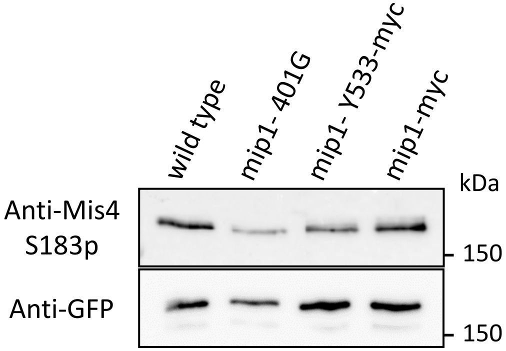

Panel C

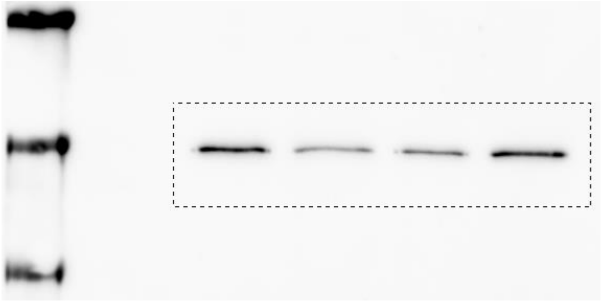

Panel C_anti-Psm1-S1022p

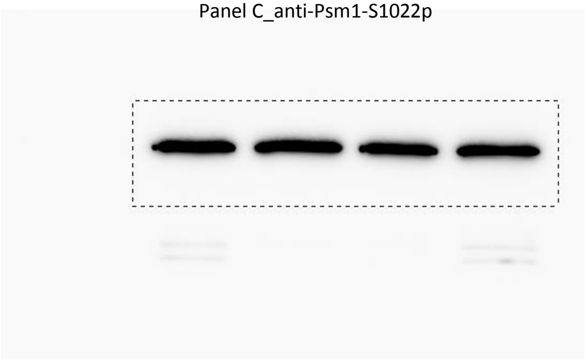

Panel C_anti-Psm1

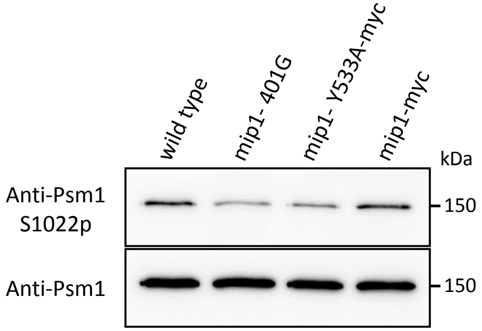

Supplement: Figure 5—figure supplement 1—source data 2. [file elife-108275-fig5-figsupp1-data2.zip › Figure 5-figure supplement 1-source data 2/Figure 5-figure supplement 1–source data 2.pdf]
