# Supplementary material for: Interplay between cohesin and TORC1 links chromosome segregation and gene expression to environmental changes
Source: eLife. 2026 Jun 1;14:RP108275. doi: 10.7554/eLife.108275 (PMC13225845; doi:10.7554/eLife.108275)

Figure 5-figure supplement 2-source data 2. The original images are on the left; the final composite is on the right.

Panel B

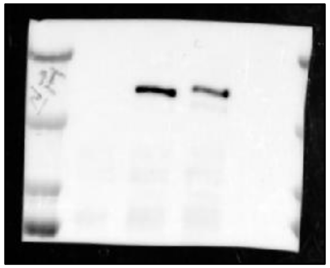

Panel B_anti-GFP

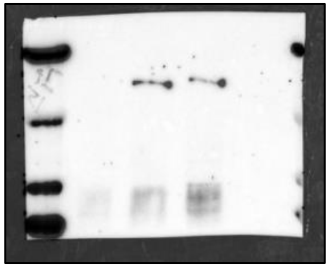

Panel B_anti Mis4-S183p

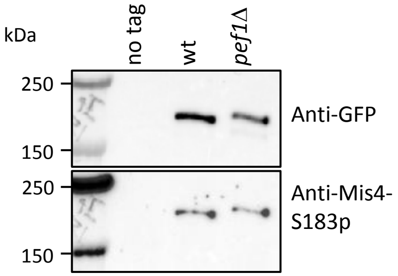

Panel D Left

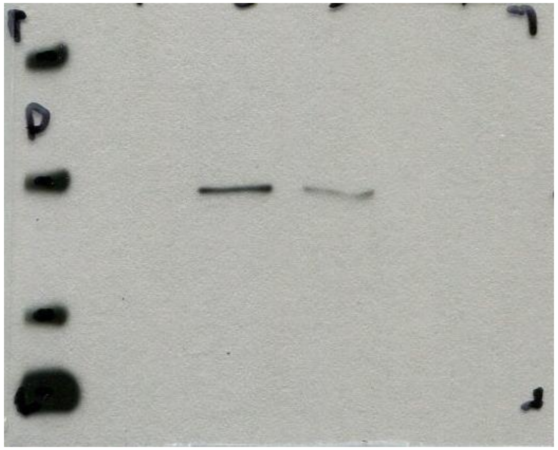

Panel D Left_anti-Psm1-S1022p

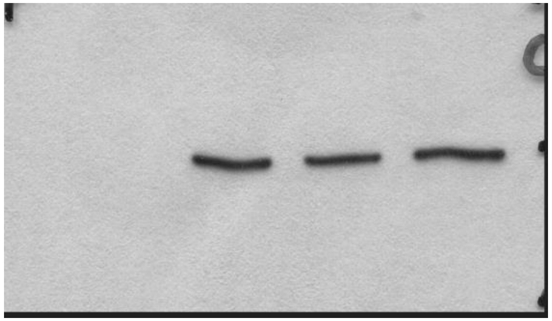

Panel D Left_anti-Psm1

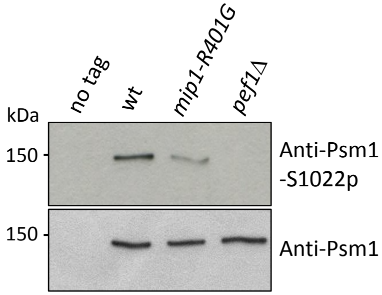

Panel D Right

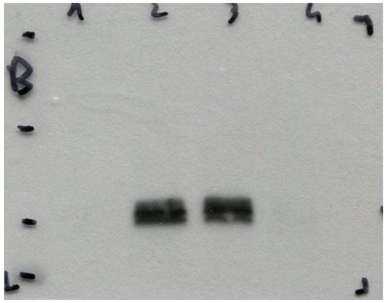

Panel D Right_anti-Rad21-T262p

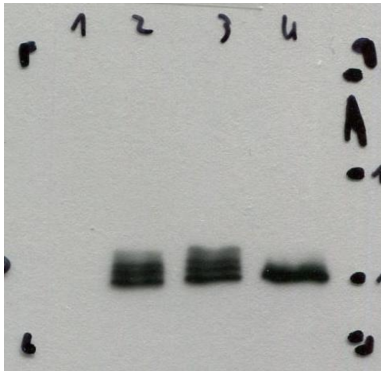

Panel D Right_anti-FLAG

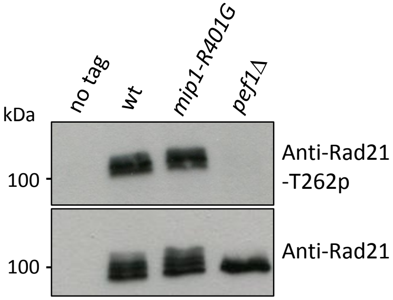

Panel E

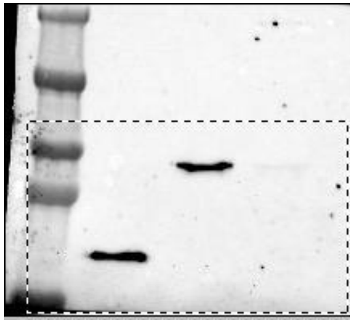

Panel E_anti-Psk1p

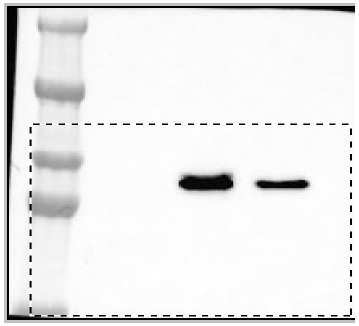

Panel E_anti-myc

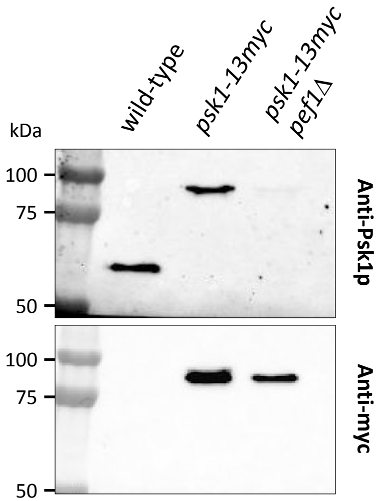

Supplement: Figure 5—figure supplement 2—source data 2. [file elife-108275-fig5-figsupp2-data2.zip › Figure 5-figure supplement 2-source data 2/Figure 5-figure supplement 2–source data 2.pdf]
