# Supplementary material for: Interplay between cohesin and TORC1 links chromosome segregation and gene expression to environmental changes
Source: eLife. 2026 Jun 1;14:RP108275. doi: 10.7554/eLife.108275 (PMC13225845; doi:10.7554/eLife.108275)

Figure 5-figure supplement 3-source data 2. The original images are on the left; the final composite is on the right.

Panel D

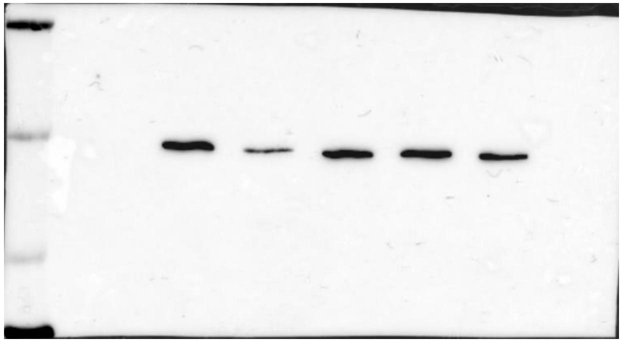

Panel D_anti-Psm1-S1022p

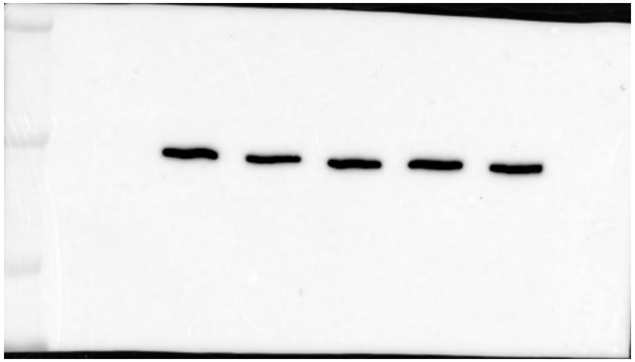

Panel D_anti-Psm1

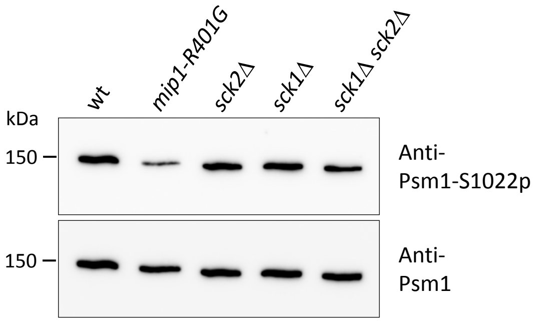

Panel E

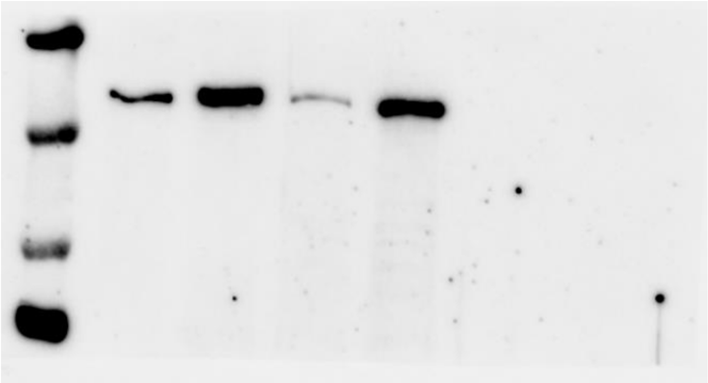

Panel E_anti-Mis4-S183p

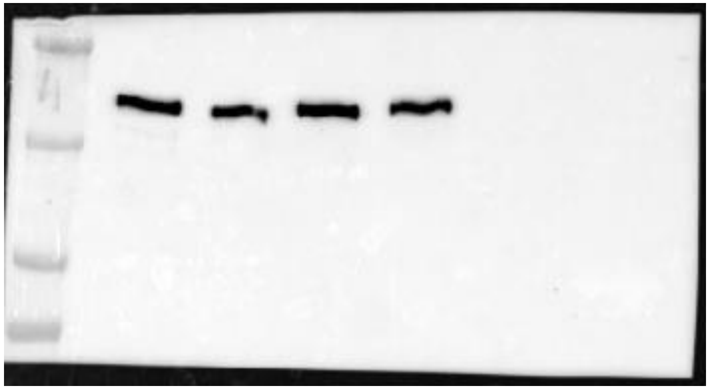

Panel E_anti-GFP

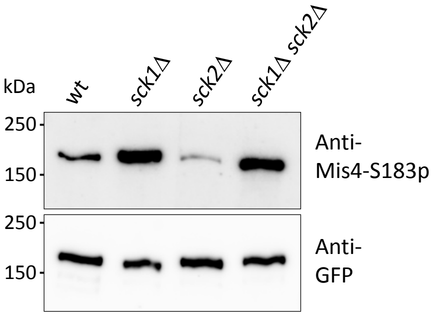

Supplement: Figure 5—figure supplement 3—source data 2. [file elife-108275-fig5-figsupp3-data2.zip › Figure 5-figure supplement 3-source data 2/Figure 5-figure supplement 3–source data 2.pdf]
